# Supplementary material for: Redox-dependent Igfbp2 signaling controls Brca1 DNA damage response to govern neural stem cell fate
Source: Nat Commun. 2023 Jan 27;14:444. doi: 10.1038/s41467-023-36174-z (PMC9883463; doi:10.1038/s41467-023-36174-z)
Supplement: Supplementary file 1 — Supplementary Information [file 41467_2023_36174_MOESM1_ESM.pdf]

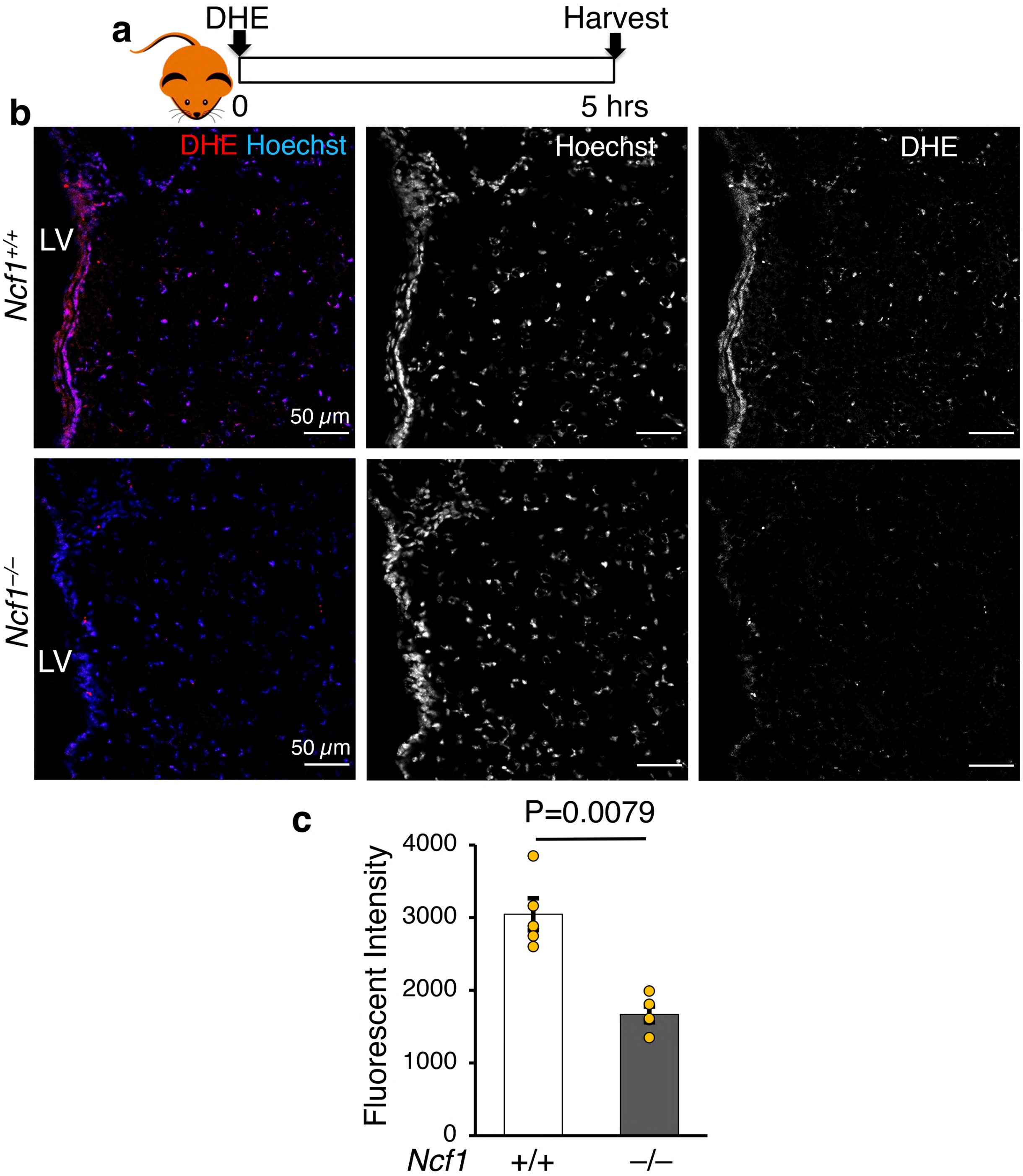

Supplementary Fig. 1: Ncf1 *in vivo* maintains the endogenous levels of reactive oxygen species (ROS) in the adult SVZ and RMS.

a, Experimental design of hydroethidine (DHE) treatment. **b**, Photomicrographs of DHE fluorescence, which is indicative of ROS levels, in the SVZ of mice. **c**, Quantification of DHE fluorescence intensity within the SVZ and RMS. $^{**}P < 0.01$, two-tailed Mann Whitney U test, n=5 mice in each group. Error bars indicate s.e.m. Scale: 50 µm. Source data are provided as a Source Data file.

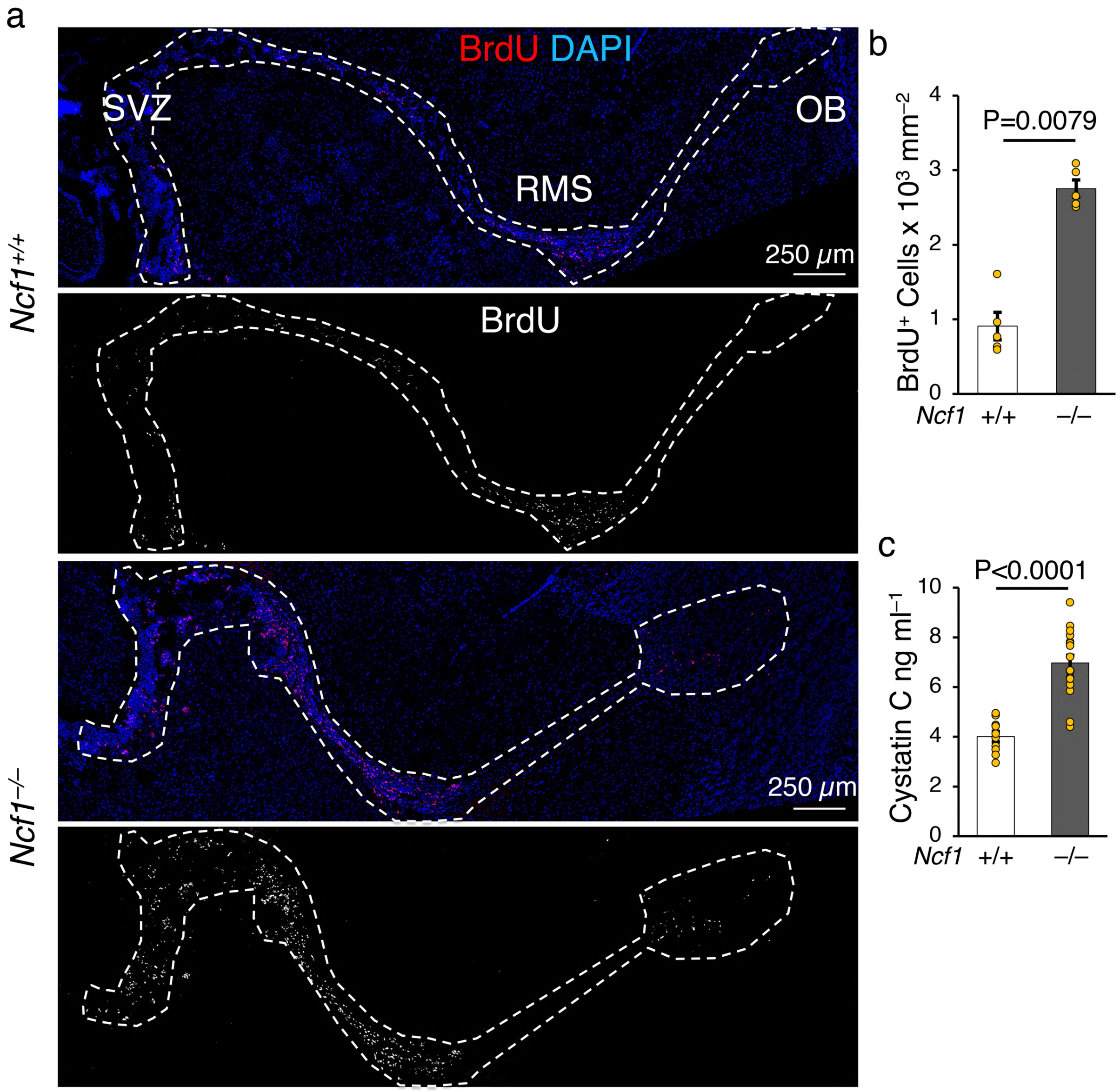

Supplementary Fig. 2: Ncf1 modulates proliferation in the adult mouse forebrain

Age matched WT and *Ncf1*$^{-/-}$ male mice received a single intraperitoneal dose of BrdU. Tissues were harvested 24 hours later. **a** Immunofluorescent staining for BrdU in parasagittal sections of the adult mouse forebrain. SVZ, subventricular zone; RMS, rostral migratory stream; OB, olfactory bulb. **b** Quantification of BrdU^{+} cells in the SVZ and RMS. (n=5 mice). **c** Quantification of cystatin-C in the mouse cerebrospinal fluid (CSF) n=10 and 18 mice in WT and *Ncf1*$^{-/-}$ groups respectively. Two-tailed Mann Whitney U test. Error bars indicate s.e.m. Scale: 250 µm. Source data are provided as a Source Data file.

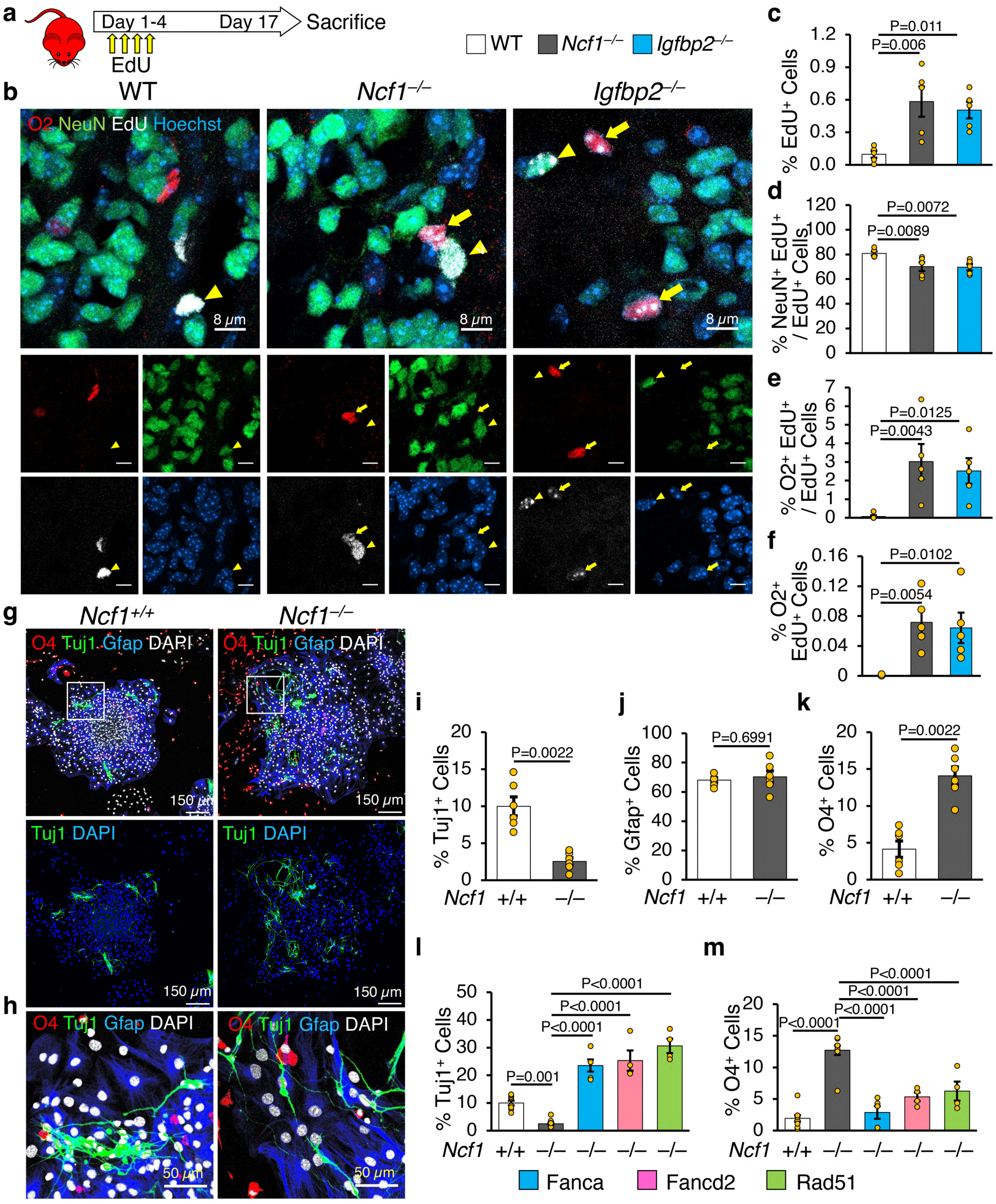

a
Day 1-4
Day 17
Sacrifice
EdU
WT
Ncf1−/−
Igfbp2−/−
b
O2 NeuN EdU Hoechst
8 μm
c
% EdU+ Cells
P=0.011
P=0.006
d
% NeuN+ EdU+ / EdU+ Cells
P=0.0072
P=0.0089
e
% O2+ EdU+ / EdU+ Cells
P=0.0125
P=0.0043
f
% O2+ EdU+ Cells
P=0.0102
P=0.0054
g
Ncf1+/+
Ncf1−/−
O4 Tuj1 Gfap DAPI
Tuj1 DAPI
150 μm
h
50 μm
i
% Tuj1+ Cells
P=0.0022
Ncf1 +/+ −/−
j
% Gfap+ Cells
P=0.6991
k
% O4+ Cells
P=0.0022
l
% Tuj1+ Cells
P<0.0001
P=0.001
Ncf1 +/+ −/− −/− −/− −/−
m
% O4+ Cells
Fanca
Fancd2
Rad51

Supplementary Fig. 3: Ncf1 alters the differentiation profile of NSCs through Igfbp2-mediated downregulation of DNA repair pathways.

a Experimental design. **b–f** Coronal sections of the OB of ten-week-old WT, *Ncf1*$^{-/-}$ and *Igfbp2*$^{-/-}$ mice from the experiment in (a) were stained for EdU (white), Oleg2 (O2) (Red), and NeuN (Green). **b** Photomicrographs of the GCL of the OB showing O2^{+}EdU^{+} cells (Yellow arrows) and NeuN^{+}EdU^{+} cells (Yellow arrow heads). **c–f** Quantification of the percentage of total cells in the GCL of the OB that were EdU^{+} (c) or O2^{+}EdU^{+} (f), and the fraction of total EdU^{+} that were double positive for NeuN^{+}EdU^{+} (d) or O2^{+}EdU^{+} (e) (n=5 mice). **g–k** Second passage WT or *Ncf1*$^{-/-}$ NSCs were grown in SFM NSC growth medium for 5 days, then cultured in differentiation medium for a further 11 days. **g** Immunofluorescent staining of representative clones for Olig 4 (O4), ßIII Tubulin (Tuj1) and Gfap. **h**, Higher magnification view of the inset in (a). **i–k** Quantification of the percentage of Tuj1^{+} (i), Gfap^{+} (j), and O4^{+} (k) cells in various groups. **l–m** WT and *Ncf1*$^{-/-}$ NSCs were transfected with 5 pmols of RNAi targeting *Fanca*, *Fancd2* or *Rad51* or negative control 3 days after plating. Quantification of Tuj1^{+} (l) and O4^{+} (m) cells at 11 days post-transfection (n=9, 10, 5, 4 and 4 donors) for *Ncf1*$^{+/+}$, *Ncf1*$^{-/-}$, *Ncf1*$^{-/-}$ treated with dsiRNA against Fanca, Fancd2 and Rad51, respectively. Kruskal-Wallis followed by Benjamini-Hochberg FDR multiple comparison posttest (c–f), two-tailed Mann Whitney U test (i–k), and One-way ANOVA followed by Bonferroni posttest (l and m). Error bars indicate s.e.m. Scale: 8 µm (b), 150 µm (g) and 50 µm (h). Source data are provided as a Source Data file.

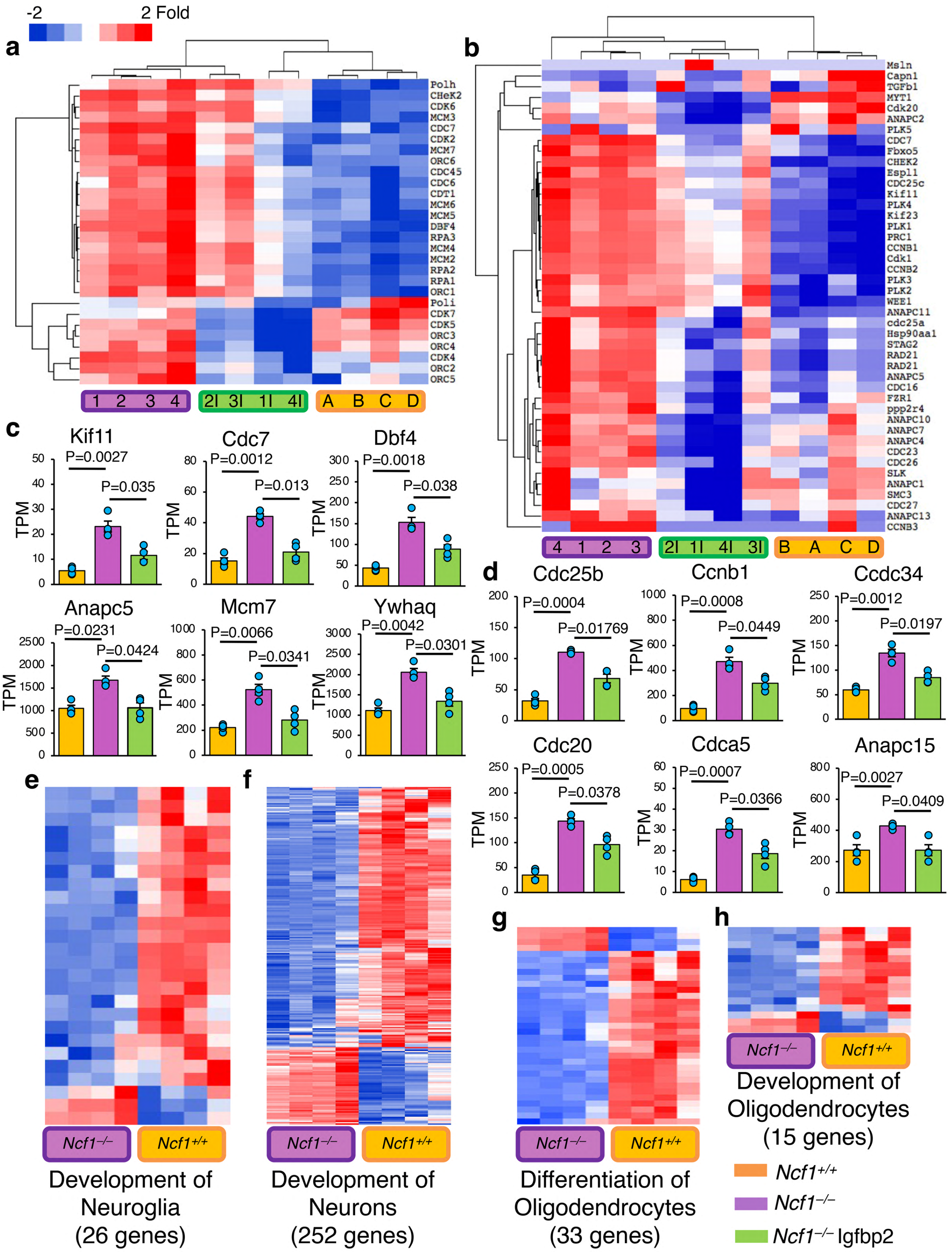

-2
2 Fold
a
Polh
CHeK2
CDK6
MCM3
CDC7
CDK2
MCM7
ORC6
CDC45
CDC6
CDT1
MCM6
MCM5
DBF4
RPA3
MCM4
MCM2
RPA2
RPA1
ORC1
Poli
CDK7
CDK5
ORC3
ORC4
CDK4
ORC2
ORC5
1 2 3 4
2I 3I 1I 4I
A B C D
b
Msln
Capn1
TGFb1
MYT1
Cdk20
ANAPC2
PLK5
CDC7
Fbxo5
CHEK2
Espl1
CDC25c
Kif11
PLK4
Kif23
PLK1
PRC1
CCNB1
Cdk1
CCNB2
PLK3
PLK2
WEE1
ANAPC11
cdc25a
Hsp90aa1
STAG2
RAD21
RAD21
ANAPC5
CDC16
FZR1
ppp2r4
ANAPC10
ANAPC7
ANAPC4
CDC23
CDC26
SLK
ANAPC1
SMC3
CDC27
ANAPC13
CCNB3
4 1 2 3
2I 1I 4I 3I
B A C D
c
Kif11
P=0.0027
P=0.035
Cdc7
P=0.0012
P=0.013
Dbf4
P=0.0018
P=0.038
Anapc5
P=0.0231
P=0.0424
Mcm7
P=0.0066
P=0.0341
Ywhaq
P=0.0042
P=0.0301
TPM
d
Cdc25b
P=0.0004
P=0.01769
Ccnb1
P=0.0008
P=0.0449
Ccdc34
P=0.0012
P=0.0197
Cdc20
P=0.0005
P=0.0378
Cdca5
P=0.0007
P=0.0366
Anapc15
P=0.0027
P=0.0409
TPM
e
f
g
h
Ncf1−/−
Ncf1+/+
Development of Neuroglia (26 genes)
Development of Neurons (252 genes)
Differentiation of Oligodendrocytes (33 genes)
Development of Oligodendrocytes (15 genes)
Ncf1+/+
Ncf1−/−
Ncf1−/− Igfbp2

Supplementary Fig. 4: Igfbp2 represses expression of cell cycle control pathways genes in *Ncf1*$^{-/-}$ NSCs and restores expression profiles to WT NSC levels

WT and *Ncf1*$^{-/-}$ NSCs were cultured for 48 hours, after which *Ncf1*$^{-/-}$ NSCs were treated with vehicle or Igfbp2. Forty-eight hours later, total RNA was collected from all groups and ribosome-depleted RNAseq was performed. **a**–**d** Ingenuity Pathway Analysis (IPA) was performed on the significantly changed gene sets in (Fig. 3a) using the absolute fold change for *Ncf1*$^{-/-}$ vs. WT and *Ncf1*$^{-/-}$ + Igfbp2 vs. *Ncf1*$^{-/-}$. This analysis revealed the top significant pathway as Cell Cycle Control of Chromosomal Replication (*Ncf1*$^{-/-}$ vs. WT *P*=2.34E-7 and *Ncf1*$^{-/-}$ + Igfbp2 vs. *Ncf1*$^{-/-}$ *P*=3.63E-10). The third most significant pathway was Mitotic Roles of Polo-Like Kinase (*Ncf1*$^{-/-}$ vs. WT *P*=2.69E-4 and *Ncf1*$^{-/-}$ + Igfbp2 vs. *Ncf1*$^{-/-}$ *P*=2.34E-6). **a**–**b** Heat maps representing clustering based on Euclidean distance of all genes in the pathways: Cell Cycle Control of Chromosomal Replication (a) and Mitotic Roles of Polo-Like Kinase (b). **c** Subset of differentially expressed genes in Cell Cycle Control of Chromosomal Replication and Mitotic Role of Polo-Like Kinase pathways. **d** Additional cell division cycle (Cdc) and anaphase-promoting complex (Anapc) genes that also significantly changed in the Figure 4A gene set. TPM: transcript per million. Benjamini-Hochberg corrected One-way ANOVA followed by Tukey's post hoc test, n=4 donors. Error bars indicate s.e.m. **e**–**h** Heat maps representing clustering based on Euclidean distance for all genes evaluated in the pathways: Development of Neuroglia, *P*=0.000822 and activation z-score of –1.45 (e), Development of Neurons, *P*=0.000944 and activation z-score of –2.219) (f), Differentiation of Oligodendrocytes, *P*=0.000685 and activation z-score of 0.289 (g), and Development of Oligodendrocytes, *P*=0.000669 (h). For a list of genes in each pathway, see Supplementary Data Table 3c. The color code for treatment groups in all heat maps and graphs is according to the legend in the lower right corner of the figure. Source data are provided as a Source Data file.

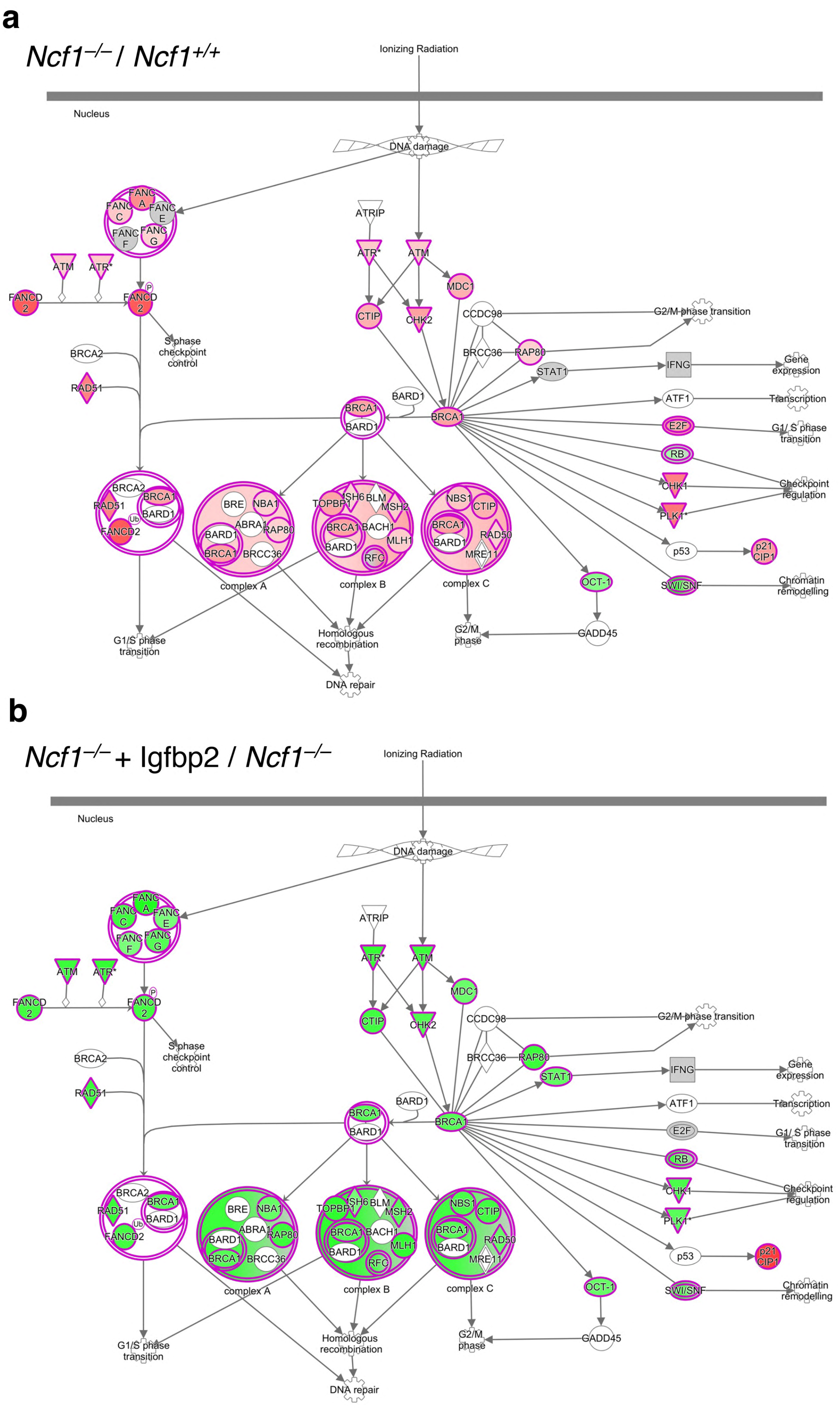
a
Ncf1−/− / Ncf1+/+
Ionizing Radiation
Nucleus
DNA damage
FANC A
FANC C
FANC E
FANC F
FANC G
ATM
ATR*
FANCD 2
FANCD 2
S phase checkpoint control
ATRIP
ATR*
ATM
MDC1
CTIP
CHK2
CCDC98
G2/M phase transition
BRCC36
RAP80
STAT1
IFNG
Gene expression
BRCA2
RAD51
BARD1
BRCA1
BARD1
BRCA1
ATF1
Transcription
E2F
G1/ S phase transition
RB
CHK1
Checkpoint regulation
PLK1*
p53
p21 CIP1
SWI/SNF
Chromatin remodelling
OCT-1
GADD45
BRCA2
BRCA1
RAD51
BARD1
FANCD2
BRE
NBA1
ABRA1
RAP80
BARD1
BRCA1
BRCC36
complex A
TOPBP1
MSH6
BLM
MSH2
BRCA1
BACH1
BARD1
MLH1
RFC
complex B
NBS1
CTIP
BRCA1
BARD1
RAD50
MRE11
complex C
G1/S phase transition
Homologous recombination
DNA repair
G2/M phase
b
Ncf1−/− + Igfbp2 / Ncf1−/−
Ionizing Radiation
Nucleus
DNA damage
FANC A
FANC C
FANC E
FANC F
FANC G
ATM
ATR*
FANCD 2
FANCD 2
S phase checkpoint control
ATRIP
ATR*
ATM
MDC1
CTIP
CHK2
CCDC98
G2/M phase transition
BRCC36
RAP80
STAT1
IFNG
Gene expression
BRCA2
RAD51
BARD1
BRCA1
BARD1
BRCA1
ATF1
Transcription
E2F
G1/ S phase transition
RB
CHK1
Checkpoint regulation
PLK1*
p53
p21 CIP1
SWI/SNF
Chromatin remodelling
OCT-1
GADD45
BRCA2
BRCA1
RAD51
BARD1
FANCD2
BRE
NBA1
ABRA1
RAP80
BARD1
BRCA1
BRCC36
complex A
TOPBP1
MSH6
BLM
MSH2
BRCA1
BACH1
BARD1
MLH1
RFC
complex B
NBS1
CTIP
BRCA1
BARD1
RAD50
MRE11
complex C
G1/S phase transition
Homologous recombination
DNA repair
G2/M phase

Supplementary Fig. 5: Ncf1 regulates the Brca1 pathway in an Igfbp2-dependent manner

WT and *Ncf1*$^{-/-}$ NSCs treated with vehicle or Igfbp2 48 hours after plating. Forty-eight hours later, total RNA was collected from all groups and ribosome-depleted RNAseq was performed. Expression data for all genes of the Brca1 pathway as absolute fold change for (*Ncf1*$^{-/-}$)/(WT) or (*Ncf1*$^{-/-}$ + Igfbp2)/(*Ncf1*$^{-/-}$) were used for Ingenuity Pathway Analysis (IPA) **a** IPA output of the Brca1 pathway comparing *Ncf1*$^{-/-}$ NSCs vs. WT NSCs (*P*=1.18E-62). **b** IPA output of Brca1 pathway comparing Igfbp2-treated *Ncf1*$^{-/-}$ NSCs vs. *Ncf1*$^{-/-}$ NSCs (*P*=3.1E-77). Green represents downregulated genes whereas red represents upregulated genes.

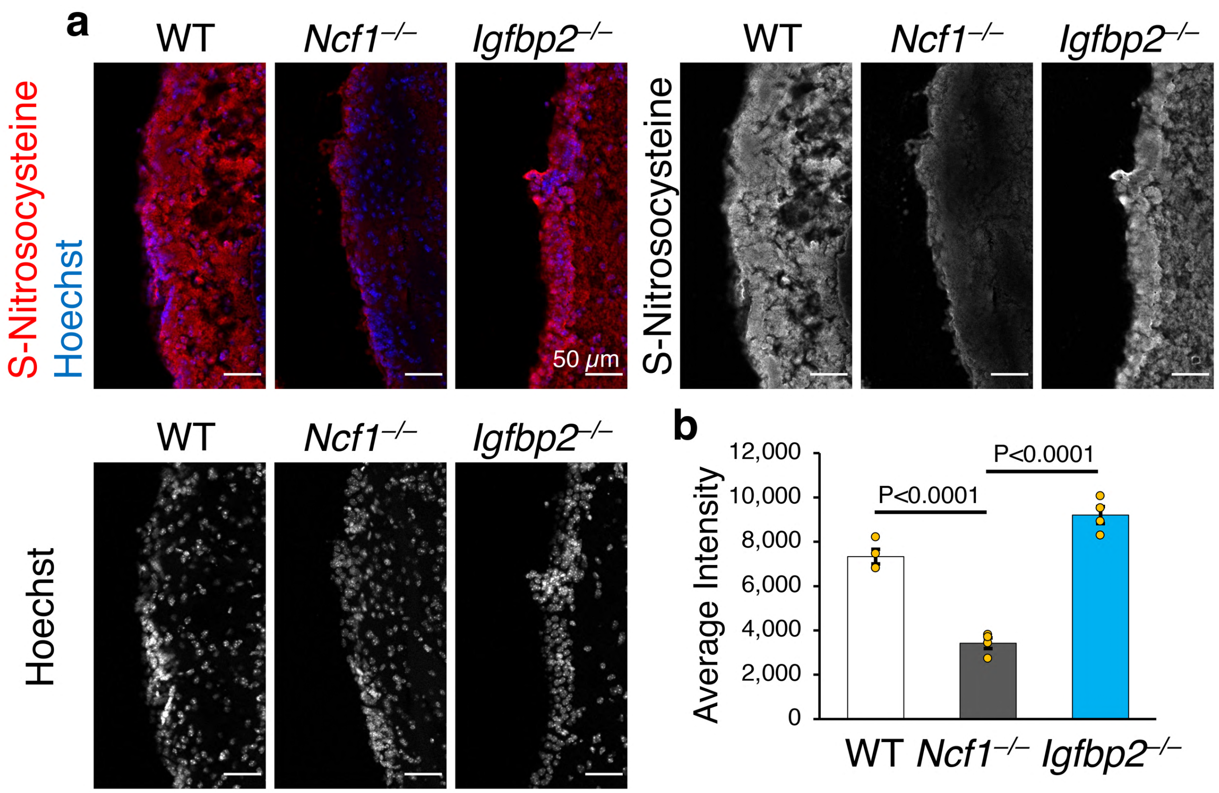

Supplementary Fig. 6: Ncf1 is required for cysteine S-Nitrosylation.
Twelve-week-old WT, *Ncf1*$^{-/-}$ and *Igfbp2*$^{-/-}$ mice were perfuse fixed and parasagittal brain sections underwent biotin derivatization to detect S-Nitrosocysteine modification. **a** Photomicrographs of S-Nitrosocysteine modification (Red) detected by biotin derivatization in the SVZ of WT, *Ncf1*$^{-/-}$ and *Igfbp2*$^{-/-}$ mice. **b** Quantification of S-Nitrosocysteine fluorescence within the SVZ (n=4 mice). ****P <0.0001, One-way ANOVA, followed by Bonferroni multiple comparison posttest. Error bars indicate s.e.m. Scale: 50 µm. Source data are provided as a Source Data file.

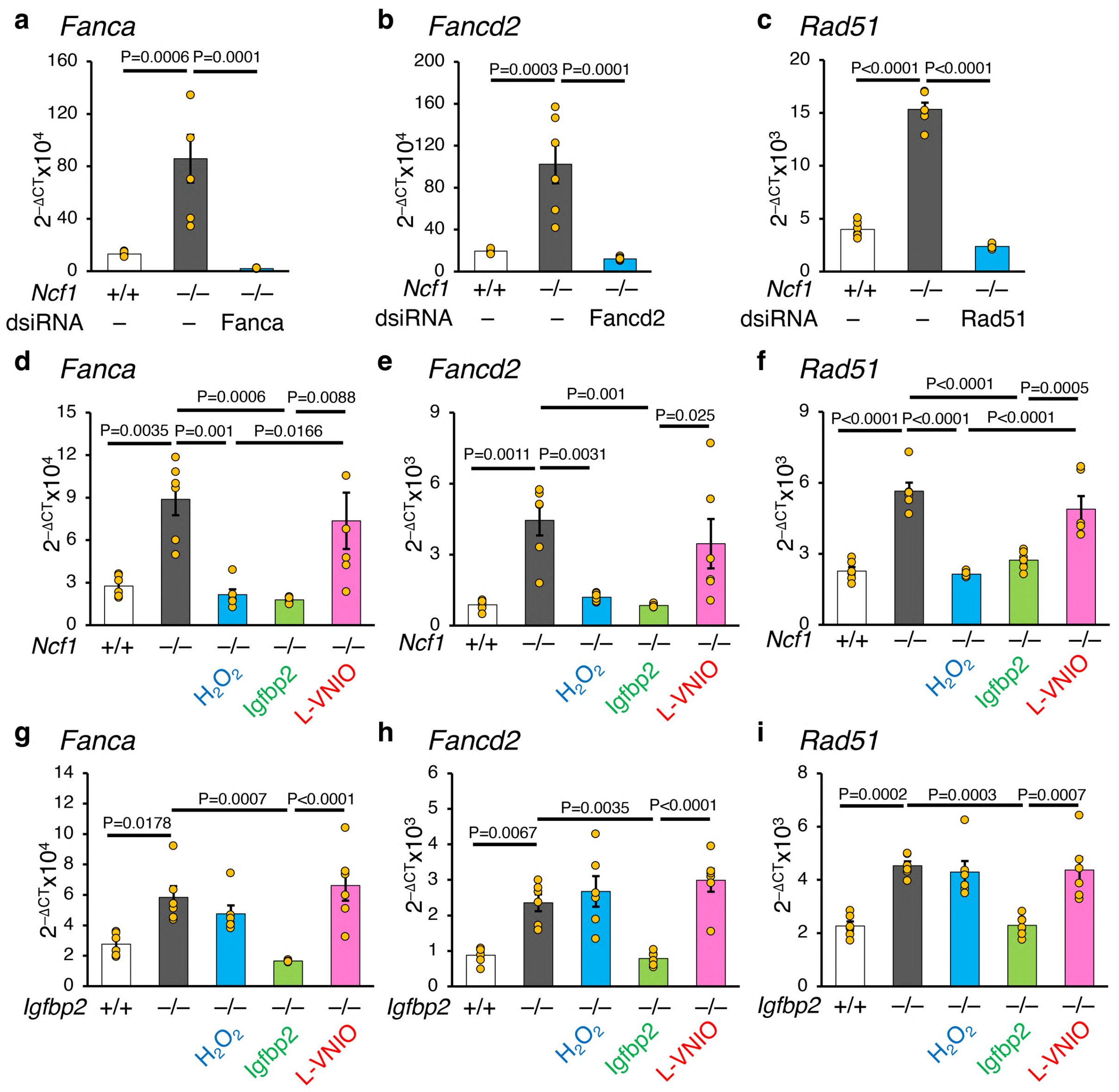

Supplementary Fig. 7: Ncf1-mediated repression of *Fanca*, *Fancd2* and *Rad51* expression is Igfbp2-dependant.

Second passage NSCs were transfected with dsiRNA against *Fanca*, *Fancd2* or *Rad51* four days after plating and harvested 24 hours after transfection (a–c). Additionally, NSCs were treated with L-VNIO for three days before harvest or treated with H_2O_2 or Igfbp2 for two days before harvest (d–i). **a–c** qPCR of *Fanca* (a), *Fancd2* (b) and *Rad51* (c) in *Ncf1*$^{-/-}$ NSCs 24 hours after transfection with dsiRNA against *Fanca*, *Fancd2* or *Rad51*, respectively. **d–f** qPCR of *Fanca* (d), *Fancd2* (e) and *Rad51* (f) in *Ncf1*$^{-/-}$ NSCs after treatment with L-VNIO, H_2O_2 or Igfbp2. **g–i** qPCR of *Fanca* (g), *Fancd2* (h) and *Rad51* (i) in *Igfbp2*$^{-/-}$ NSCs after treatment with L-VNIO, H_2O_2 or Igfbp2. ΔCT: the difference in threshold cycles normalized to β-Actin. n=6 donors for all groups. One-way ANOVA followed by Bonferroni multiple comparison posttest (i and j). Error bars indicate s.e.m. Source data are provided as a Source Data file.

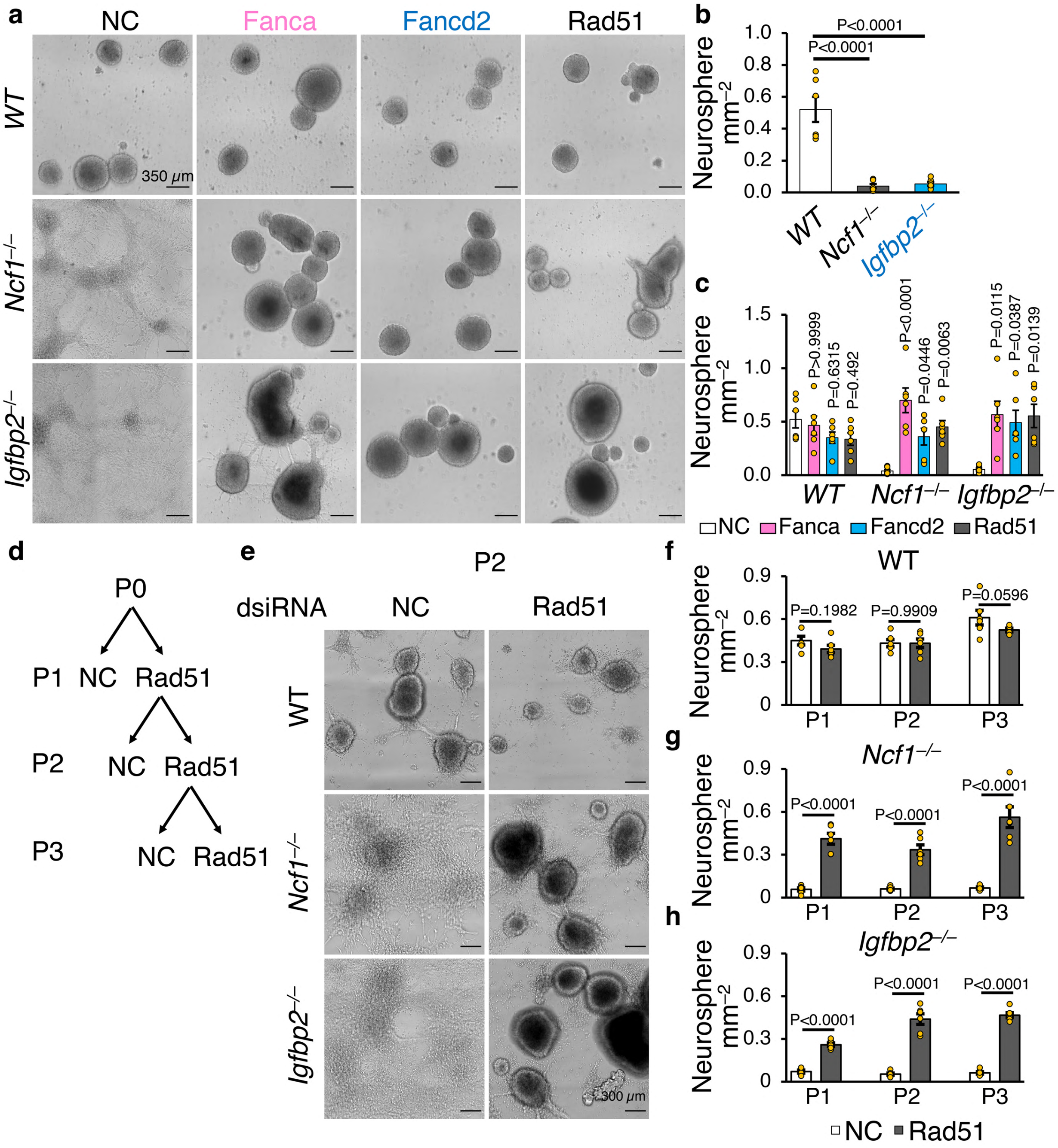

a
NC
Fanca
Fancd2
Rad51
WT
Ncf1–/–
Igfbp2–/–
350 μm
b
Neurosphere mm−2
P<0.0001
P<0.0001
WT
Ncf1–/–
Igfbp2–/–
c
Neurosphere mm−2
P>0.9999
P=0.6315
P=0.492
P<0.0001
P=0.0446
P=0.0063
P=0.0115
P=0.0387
P=0.0139
WT
Ncf1–/–
Igfbp2–/–
NC Fanca Fancd2 Rad51
d
P0
P1 NC Rad51
P2 NC Rad51
P3 NC Rad51
e
P2
dsiRNA NC Rad51
WT
Ncf1–/–
Igfbp2–/–
300 μm
f
WT
Neurosphere mm−2
P=0.1982
P=0.9909
P=0.0596
P1 P2 P3
g
Ncf1–/–
Neurosphere mm−2
P<0.0001
P<0.0001
P<0.0001
P1 P2 P3
h
Igfbp2–/–
Neurosphere mm−2
P<0.0001
P<0.0001
P<0.0001
P1 P2 P3
NC Rad51

Supplementary Fig. 8: Components of the Brca1 DNA damage response pathway regulate neurosphere formation in NSCs.

a–**c** Second passage SVZ ANSCs from eight-week-old WT, *Ncf1*$^{-/-}$ or *Igfbp2*$^{-/-}$ mice were transfected with negative control (NC) dsiRNA or dsiRNA against *Fanca*, *Fancd2* or *Rad51* three days after plating. **a** Photomicrographs of NSCs 11 days after transfection. **b**–**c** Quantification of density of neurospheres ≥60 µm in diameter within NC-transfected ANSCs (b) and all transfected groups (c), n=6 donors. **d** Experimental design for passages (P1, P2 and P3) of NSCs and transfection with control (NC) dsiRNA or *Rad51* dsiRNA after each passage. **e** Photomicrographs of second passage (P2) NSCs 11 days after transfection. **f**–**h** Quantification of neurosphere ≥ 60 µm in diameter in WT (f), *Ncf1*$^{-/-}$ (g) and *Igfbp2*$^{-/-}$ (h) NSCs, n=6 donors. One-way ANOVA followed by Bonferroni multiple comparison posttest (b–c) and Two-way ANOVA followed by Original FDR method of Benjamini and Hochberg multiple comparison posttest (f–h). Error bars indicate s.e.m. Scale: 350 µm (a) and 300 µm (e). Source data are provided as a Source Data file.

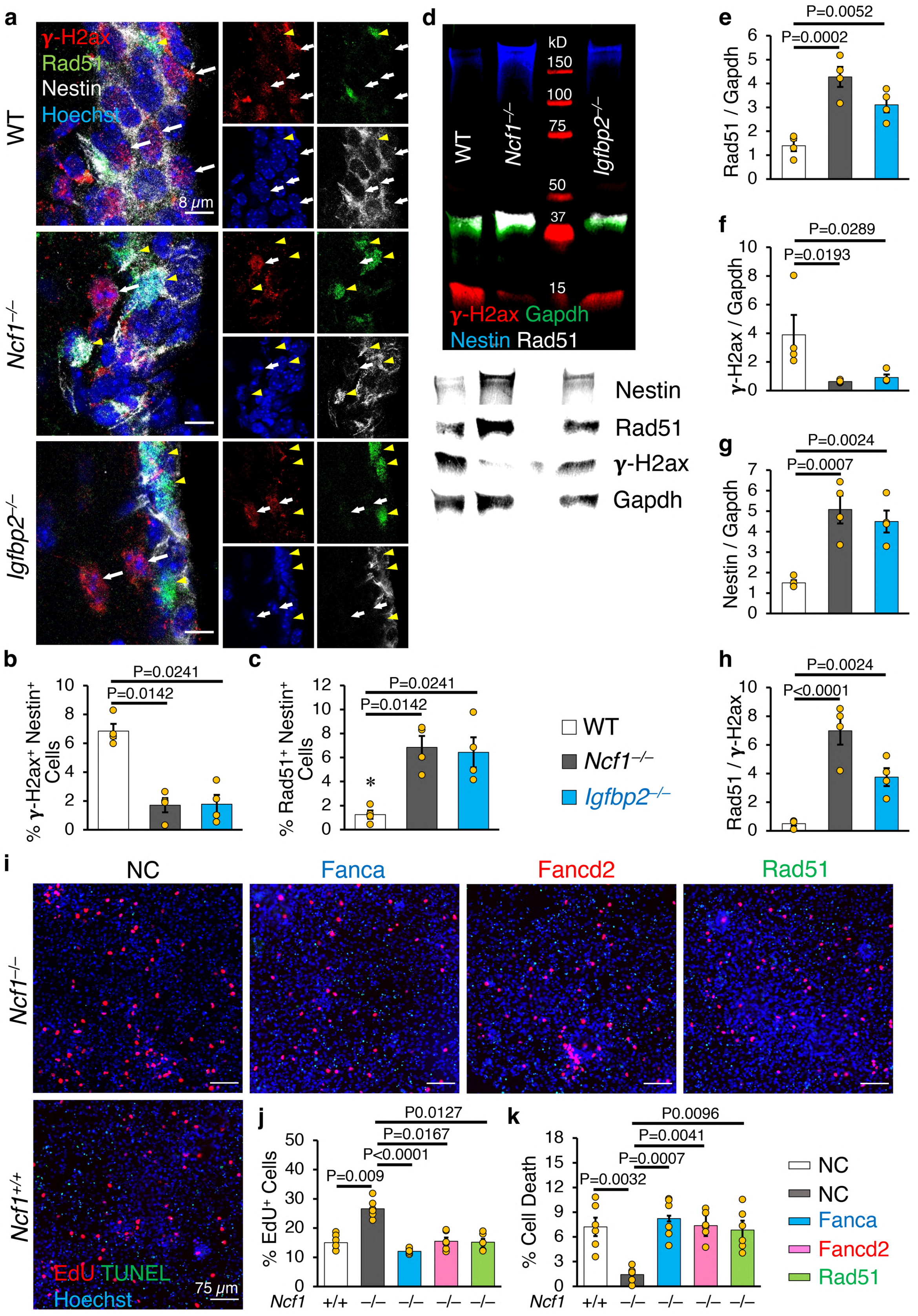

a
γ-H2ax
Rad51
Nestin
Hoechst
WT
8 μm
Ncf1−/−
Igfbp2−/−
d
kD
150
100
75
50
37
15
WT
Ncf1−/−
Igfbp2−/−
γ-H2ax Gapdh
Nestin Rad51
Nestin
Rad51
γ-H2ax
Gapdh
e
Rad51 / Gapdh
P=0.0052
P=0.0002
f
γ-H2ax / Gapdh
P=0.0289
P=0.0193
g
Nestin / Gapdh
P=0.0024
P=0.0007
h
Rad51 / γ-H2ax
P=0.0024
P<0.0001
b
% γ-H2ax+ Nestin+ Cells
P=0.0241
P=0.0142
c
% Rad51+ Nestin+ Cells
P=0.0241
P=0.0142
*
WT
Ncf1−/−
Igfbp2−/−
i
NC
Fanca
Fancd2
Rad51
Ncf1−/−
Ncf1+/+
EdU TUNEL
Hoechst
75 μm
j
% EdU+ Cells
P0.0127
P=0.0167
P<0.0001
P=0.009
Ncf1 +/+ −/− −/− −/− −/−
k
% Cell Death
P0.0096
P=0.0041
P=0.0007
P=0.0032
Ncf1 +/+ −/− −/− −/− −/−
NC
NC
Fanca
Fancd2
Rad51

Supplementary Fig. 9: Ncf1 regulates NSC proliferation/death through Igfbp2-dependant regulation of DNA repair network.
a–**c** Ten-week-old WT, *Ncf1*$^{-/-}$ and *Igfbp2*$^{-/-}$ mice were perfuse fixed and coronal brain sections were stained by immunofluorescence for Rad51 in green (yellow arrow heads), γ-H2ax in red (white arrows), and Nestin (white). **a** Photomicrographs of coronal sections of the SVZ of WT, *Ncf1*$^{-/-}$ and *Igfbp2*$^{-/-}$ mice. **b**–**c** Quantification of γ-H2ax^{+} Nestin^{+} (b) and Rad51^{+} Nestin^{+} (c) cells (n=4 animals). **d**–**h** Whole membrane and single channels western blot (d) and quantification of Rad51 (e), γ-H2ax (f), Nestin (g) levels (relative to Gapdh) and Rad51 levels relative to γ-H2ax (h) in NSC lysates five days after plating (n=4 donors). The samples were derived from the same experiment and blots were processed in parallel. kD=kilodalton. **i**–**k** Second passage WT and *Ncf1*$^{-/-}$ NSCs were transfected with negative control (NC) dsiRNA or dsiRNA against *Fanca*, *Fancd2* or *Rad51* three days after plating. 48 hours later NSCs were pulsed with EdU four hours before fixation. **i** Photomicrographs of WT and *Ncf1*$^{-/-}$ NSCs labelled for EdU (Red) and cell death (Green). **j**–**k** quantification of EdU^{+} (j) and TUNEL^{+} (k) cells (n=6 donors). Kruskal-Wallis (b–c and j–k), and one-way ANOVA (e–h) followed by Benjamini-Hochberg FDR multiple comparison posttest. Error bars indicate s.e.m. Scale: 8 µm (a) and 75 µm (d). Source data are provided as a Source Data file.

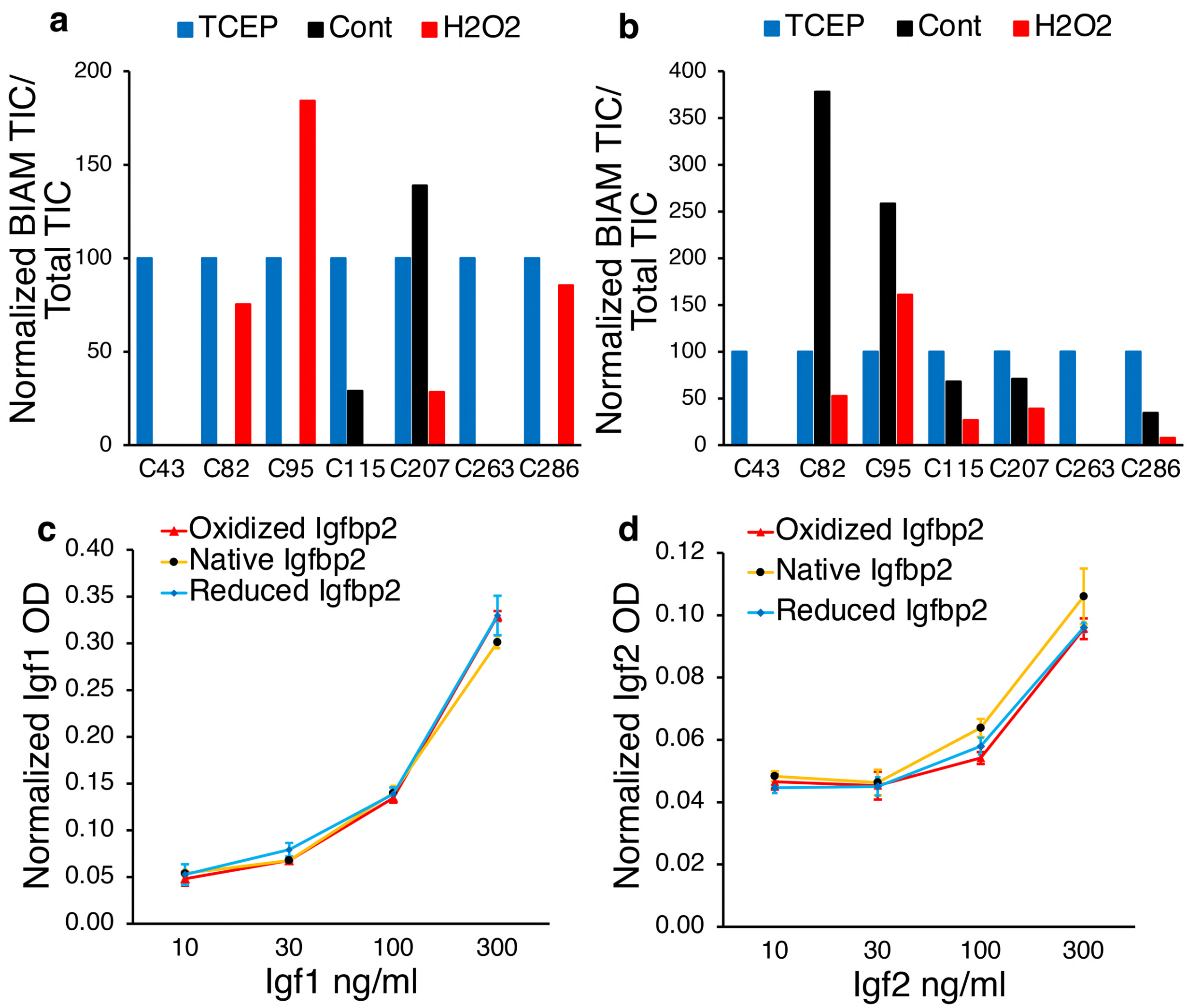

Supplementary Fig. 10: Igfbp2 redox sensitive cysteines 43 and 263 carry free SH groups only under reducing conditions.

a–**b** Thiol groups on native, reduced (TCEP-pretreated) and Oxidized (H_2O_2-pretreated) recombinant mouse Igfbp2 were labelled with BIAM then run on LC-MS/MS (See experimental design in Fig. 4a). Results of two independent experiments are shown. **c**–**d** Native, reduced (DTT-pretreated) or oxidized (H_2O_2-pretreated) mouse recombinant Igfbp2 was incubated with increasing concentrations of biotinylated recombinant Igf1 (c) or Igf2 (d) for 18 hours at 4˚C. The reaction was then incubated for 2 hours at room temperature in an ELISA plate coated with anti-Igfbp2 antibody. Then Igfbp2-bound biotinylated Igfs were detected with HRP-conjugated streptavidin. Two-way ANOVA followed by Bonferroni multiple comparison posttest. n=4 from two independent experiments. Error bars indicate s.e.m. Source data are provided as a Source Data file.
