# Supplementary material for: Redox-dependent Igfbp2 signaling controls Brca1 DNA damage response to govern neural stem cell fate
Source: Nat Commun. 2023 Jan 27;14:444. doi: 10.1038/s41467-023-36174-z (PMC9883463; doi:10.1038/s41467-023-36174-z)
Supplement: Supplementary file 3 — Description of Additional Supplementary Files [file 41467_2023_36174_MOESM3_ESM.pdf]

Description of Additional Supplementary Files

Supplementary Data 1: Peptide quantitative report of LC-MS/MS experiment comparing the secretome of WT to that of *Ncf1*$^{-/-}$ NSCs. Conditioned media of WT and *Ncf1*$^{-/-}$ NSCs were collected five days after plating, digested with trypsin and sequentially ran on LTQ liquid chromatography mass spectrometer. The resultant traces were searched using Mascot search engine for protein identification. Scaffold software was used to visualize and compare the two secretomes.

Supplementary Data 2a.: RNAseq quantification of WT, *Ncf1*$^{-/-}$, and *Ncf1*$^{-/-}$ + Igfbp2 NSC transcriptomes. RNAseq on rRNA-depleted RNA from NSC cultures. Median quality for all bases was equivalent for all samples; the median phred score qualities for each base was ≥ 32. Samples were then mapped with Bowtie2 and quantified with RSEM. Transcripts per million (TPM) were the units used in all downstream analysis. The genes are listed in rank order according to significance using FDR *q*-value for differences in abundance between WT, *Ncf1*$^{-/-}$ and/or *Ncf1*$^{-/-}$ + Igfbp2 samples; One-Way ANOVA, Benjamini-Hochberg (BH) adjusted and Tukey posttest for comparisons of groups.

Supplementary Data 2b.: Significantly changed transcripts in WT, *Ncf1*$^{-/-}$, and *Ncf1*$^{-/-}$ + Igfbp2 NSC transcriptomes. The genes from Supplementary Data Table 2a that were significantly differed between WT, *Ncf1*$^{-/-}$, and/or *Ncf1*$^{-/-}$ + Igfbp2 samples (*P*-value < 0.05) are listed in rank order according to the significance of their FDR *q*-value for differences in abundance between WT, *Ncf1*$^{-/-}$ and/or *Ncf1*$^{-/-}$ + Igfbp2 samples. One-Way ANOVA, Benjamini-Hochberg (BH) adjusted and Tukey posttest was used for comparisons of groups. This list of genes was used to generate heat maps in Fig. 3a.

Supplementary Data 2c.: Significantly changed transcripts in *Ncf1*$^{-/-}$ vs. WT NSC transcriptomes. The genes from Supplementary Data Table 2b that significantly differed between WT and *Ncf1*$^{-/-}$ samples (*P*-value < 0.05), by One-Way ANOVA, Benjamini-Hochberg (BH) adjusted, Tukey posttest, are listed in rank order according to the significance of their FDR *q*-value for differences in abundance between WT vs. *Ncf1*$^{-/-}$. Values of those genes for *Ncf1*$^{-/-}$ + Igfbp2 samples are also listed for comparison regardless of their significance. This list of genes was used to generate heat maps in Fig. 3b.

Supplementary Data 2d.: *Ncf1*$^{-/-}$-induced NSC transcripts that significantly changed toward WT levels after Igfbp2 treatment. The genes from Supplementary Data Table 2c that significantly differed between *Ncf1*$^{-/-}$ and *Ncf1*$^{-/-}$ + Igfbp2 samples (*P*-value < 0.05), but were not significantly different between *Ncf1*$^{-/-}$ + Igfbp2 and WT samples (*P*-value ≥ 0.05) by One-Way ANOVA, Benjamini-Hochberg (BH) adjusted, Tukey posttest, are listed in rank order according to the significance of their FDR *q*-value for differences in abundance between WT, *Ncf1*$^{-/-}$, and *Ncf1*$^{-/-}$ + Igfbp2 samples. This list of genes was used to generate heat maps in Fig. 3c.

Supplementary Data 3a.: Canonical pathways from NSC RNAseq data for all significantly changed genes. The absolute fold change in gene expression for *Ncf1*$^{-/-}$ + Igfbp2 vs. *Ncf1*$^{-/-}$ and *Ncf1*$^{-/-}$ vs. WT were subjected to Ingenuity Pathway Analysis (IPA, Qiagen). All genes that significantly differed by One-Way ANOVA, Benjamini-Hochberg (BH) adjusted (Supplementary Data Table 2b) were used in two separate IPA analyses using the fold change for the two comparisons. Shown are the list of identified canonical pathways for both comparisons – log(*p*-value), *p*-value, z-score and the list of genes identified in each canonical pathway. Only pathways that were significant (*P*<0.05) in one or both comparisons are listed.

Supplementary Data 3b.: Canonical pathways from NSC RNAseq data for the subset of Ncf1/Igfbp2-dependent genes. The absolute fold change in gene expression for *Ncf1*$^{-/-}$ + Igfbp2 vs. *Ncf1*$^{-/-}$ and *Ncf1*$^{-/-}$ vs. WT were subjected to Ingenuity Pathway Analysis (IPA, Qiagen). Only genes that significantly differed by One-Way ANOVA, Benjamini-Hochberg (BH) adjusted between *Ncf1*$^{-/-}$ + Igfbp2 vs. *Ncf1*$^{-/-}$, but not *Ncf1*$^{-/-}$ + Igfbp2 vs. WT (Supplementary Data Table 2d), were used in IPA

analysis using the fold change in gene expression for the two comparisons. Shown are the list of identified canonical pathways for both comparisons – log(*p*-value), *p*-value, z-score and the list of genes identified in each canonical pathway. Only pathways that were significant (P<0.05) in one or both comparisons are listed.

Supplementary Data 3c.: Diseases and functions pathways from NSC RNAseq data for all significantly changed genes. The absolute fold change in gene expression for *Ncf1*$^{-/-}$ + Igfbp2 vs. *Ncf1*$^{-/-}$ and *Ncf1*$^{-/-}$ versus WT were subjected to Ingenuity Pathway Analysis (IPA, Qiagen). All genes that significantly differed by One-Way ANOVA, Benjamini-Hochberg (BH) adjusted (Supplementary Data Table 2b) were used in two separate IPA analyses using the fold change for the two comparisons. Shown are the list of identified diseases and functions pathways for both comparisons, *p*-values, and the list of genes identified in each pathway.

Supplementary Software 1: RNAseq Analysis Code. The zip package contains an R script titled "merge_a nnotate_TPM_bulk_Weam_2.R" that performs all differential expression analysis. The inputs for analysis are contained within the "inputs" directory; the expected outputs are contained within the "expected_outp uts.zip" file.
