# Supplementary material for: Redox-dependent Igfbp2 signaling controls Brca1 DNA damage response to govern neural stem cell fate
Source: Nat Commun. 2023 Jan 27;14:444. doi: 10.1038/s41467-023-36174-z (PMC9883463; doi:10.1038/s41467-023-36174-z)
Supplement: Supplementary file 8 — Reporting Summary [file 41467_2023_36174_MOESM8_ESM.pdf]

nature research

Corresponding author(s): John F. Engelhardt

Last updated by author(s): Dec 28, 2022

Reporting Summary

Nature Research wishes to improve the reproducibility of the work that we publish. This form provides structure for consistency and transparency in reporting. For further information on Nature Research policies, see our Editorial Policies and the Editorial Policy Checklist.

Statistics

For all statistical analyses, confirm that the following items are present in the figure legend, table legend, main text, or Methods section.

n/a	Confirmed	
☐	☒	The exact sample size (n) for each experimental group/condition, given as a discrete number and unit of measurement
☐	☒	A statement on whether measurements were taken from distinct samples or whether the same sample was measured repeatedly
☐	☒	The statistical test(s) used AND whether they are one- or two-sided<br>*Only common tests should be described solely by name; describe more complex techniques in the Methods section.*
☐	☒	A description of all covariates tested
☐	☒	A description of any assumptions or corrections, such as tests of normality and adjustment for multiple comparisons
☐	☒	A full description of the statistical parameters including central tendency (e.g. means) or other basic estimates (e.g. regression coefficient) AND variation (e.g. standard deviation) or associated estimates of uncertainty (e.g. confidence intervals)
☐	☒	For null hypothesis testing, the test statistic (e.g. F, t, r) with confidence intervals, effect sizes, degrees of freedom and P value noted<br>*Give P values as exact values whenever suitable.*
☒	☐	For Bayesian analysis, information on the choice of priors and Markov chain Monte Carlo settings
☐	☒	For hierarchical and complex designs, identification of the appropriate level for tests and full reporting of outcomes
☒	☐	Estimates of effect sizes (e.g. Cohen's d, Pearson's r), indicating how they were calculated

Our web collection on statistics for biologists contains articles on many of the points above.

Software and code

Policy information about availability of computer code

Data collection	ImgeStudio version 4.0.21 (Li-cor Bioscience). Zen (blue edition), version 3.4.91.00000 (Carl Zeiss Inc, NY). MetaMorph version 7.10.3.279 (Molecular Devices, CA). Mascot Version 2.4.0 (Matrix Science)
Data analysis	GraphPad Prism (San Diego, CA)(7.0.0). FIJI (https://fiji.sc/) (ImageJ 1.53c). Excel (Office 365). MetaMorph version 7.10.3.279 (Molecular Devices, CA). ImageStudioLite virsion 4.0.21 and Empiria Studio version 2.3.0.154 (Li-cor Bioscience). Ingenuity Pathway Analysis (IPA) version 01-19-00 (Qiagen). Bowtie2 version 2.3.4.2 (Johns Hopkins University). RNA-Seq by Expectation-Maximization (RSEM) version v1.2.30. Scaffold Version 4.3.2 (Proteome Software Inc.). The custom code package used for RNAseq data analysis (Scott RNAseq analysis code) is included as a supplementary data zip file with the paper.

For manuscripts utilizing custom algorithms or software that are central to the research but not yet described in published literature, software must be made available to editors and reviewers. We strongly encourage code deposition in a community repository (e.g. GitHub). See the Nature Research guidelines for submitting code & software for further information.

Data

Policy information about availability of data

All manuscripts must include a data availability statement. This statement should provide the following information, where applicable:

- Accession codes, unique identifiers, or web links for publicly available datasets
- A list of figures that have associated raw data
- A description of any restrictions on data availability

All original RNA sequencing data reported in this paper are publicly available on the NCBI Gene Expression Omnibus (GEO) (https://www.ncbi.nlm.nih.gov/geo/) under the accession code GSE221955. A reporting summary for this article is available as a supplementary information file. The main data supporting the findings of this study are available within the article and its Supplementary Figures and supplementary data tables. Source data are provided with this paper. Complete

statistics and exact P values for each test and post hoc comparison are also included within the source data. Additional details on datasets and protocols that support the findings of this study will be made available by the corresponding author upon reasonable request.

Field-specific reporting

Please select the one below that is the best fit for your research. If you are not sure, read the appropriate sections before making your selection.

- [x] Life sciences
- [ ] Behavioural & social sciences
- [ ] Ecological, evolutionary & environmental sciences

For a reference copy of the document with all sections, see nature.com/documents/nr-reporting-summary-flat.pdf

Life sciences study design

All studies must disclose on these points even when the disclosure is negative.

Sample size	Sample size was based on previous experience and published data that utilized non-adherent and mono-layer neural stem cell cultures. This was according to the University of Iowa IACUC recommendations of bringing the use of experimental animals to the minimum.
Data exclusions	No data were excluded from these analyses
Replication	All attempts at replication were successful. Animals from multiple independent litters were used for each in vivo experiment. All experiments involving primary mouse neural stem cells were replicated on isolates from separate donors (i.e. different animals) from at least three individual donors.
Randomization	All mice, wild type and transgenic, were treated the exact same way and randomly assigned to different groups in all in vivo experiments. For in vitro experiments, cells from each donor group were plated into multiple same area vessels at the same density. Vessels from each donor group with the same plating density were randomly assigned to control or treated groups (eg. dsiRNA, Igfbp2, plasmid, PeggyBac or L VNIO treated groups).
Blinding	Blinding for neurosphere counting was not possible due to gross differences between genotypes. Neurosphere and immunofluorescence based marker quantification was automated using MetaMorph with the same settings applied across various groups and samples. Blinding was not relevant for experiments involving unbiased automated counts (i.e. ELISA). For all other experiments, investigators were blinded of group allocation during data collection and analysis.

Reporting for specific materials, systems and methods

We require information from authors about some types of materials, experimental systems and methods used in many studies. Here, indicate whether each material, system or method listed is relevant to your study. If you are not sure if a list item applies to your research, read the appropriate section before selecting a response.

Materials & experimental systems

n/a	Involved in the study	
[ ]	[x]	Antibodies
[x]	[ ]	Eukaryotic cell lines
[x]	[ ]	Palaeontology and archaeology
[ ]	[x]	Animals and other organisms
[x]	[ ]	Human research participants
[x]	[ ]	Clinical data
[x]	[ ]	Dual use research of concern

Methods

n/a	Involved in the study	
[x]	[ ]	ChIP-seq
[x]	[ ]	Flow cytometry
[x]	[ ]	MRI-based neuroimaging

Antibodies

Antibodies used

Primary antibodies:

Chicken polyclonal anti-Nestin, (Novus Biologicals, Cat# NB100-1604, RRID:AB_2282642, 1:250 dilution)

Purified Mouse monoclonal anti-BrdU, clone B44, (BD Biosciences Cat# 347580, RRID:AB_10015219, 1:250 dilution)

Rabbit polyclonal anti-NeuN, (Abcam, Cat# ab104225, RRID:AB_10711153, 1:400 dilution)

Chicken polyclonal anti-GFAP (Aves labs Cat# GFAP, RRID:AB_2313547, 1:250 dilution)

Mouse monoclonal anti-O2, clone 211F1.1 (Millipore, Cat# MABN50, RRID:AB_10807410, 1:500 dilution)

Mouse monoclonal anti-flag, clone M2, (Sigma Aldrich, Cat# F1804, RRID:AB_262044, 1:500 dilution)

Rabbit polyclonal anti-Igfbp2, (Millipore, Cat# 06-107, RRID:AB_310049, 1:1000 dilution)

Goat polyclonal anti-Igfbp2, (R&D SYSTEMS, Cat# AF797,RRID:AB_355609, 1:1000 dilution)

Rabbit monoclonal anti-gamma H2AX (phospho S139), clone: EP854(2)Y, (Abcam, Cat# ab81299, RRID:AB_1640564, 1:1000 dilution)

Goat polyclonal anti-Gapdh, (Thermo Fisher Scientific, Cat# PA1-9046, RRID:AB_1074703, 1:1000 dilution)

Mouse polyclonal anti-Rad51, (Abcam, Cat# ab88572, RRID:AB_2042762, 1:330 dilution)

Validation

All of the antibodies used in the manuscript are all commercially available. All the antibodies used here have been well characterized and validated by the providers or previous publications. Below are the providers' links to the antibody information and relevant publications.

Anti-Nestin antibody. From the manufacturer: We have publications tested in 4 confirmed species: Human, Mouse, Rat, Monkey. We have publications tested in 6 applications: Flow, ICC/IF, IHC, IHC-Fr, IHC-P, WB. (https://www.novusbio.com/products/nestin-antibody_nb100-1604#reviews-publications). (Platel et al., 2009, Glia. 2009 Jan 1;57(1):66-78).

Anti-BrdU antibody. From the manufacturer: Anti-BrdU identifies BrdU (but not thymidine) in single-stranded DNA, free BrdU, or BrdU coupled to a protein carrier. The antibody also reacts with iodouridine (https://www.bdbiosciences.com/us/applications/research/apoptosis/purified-antibodies/purified-mouse-anti-brdu-b44/p/347580). (Gratzner et al., Science. 1982;218:474).

Anti-NeuN antibody. From the manufacturer: Tested applications suitable for: WB, IHC-FrFl, IHC-P, IHC-FoFrmore. Reacts with: Mouse, Rat, Human. (https://www.abcam.com/neun-antibody-neuronal-marker-ab104225.html). (Macchi et al., 2020, Elife 9:N/A (2020))

Anti-GFAP antibody. From the manufacturer: This antipeptide IgY antibody was generated in chickens against a C-terminal sequence shared between the mouse (NP_032109) and rat (NP_05889.1) gene products. It has been validated with human, mouse and rat tissues. (https://www.aveslabs.com/products/glial-fibrillary-acidic-protein-gfap?_pos=3&_sid=01c0781be&_ss=r). (Szot et al., 2018, Neuroscience. 10.1016/j.neuroscience.2017.04.028)

Anti-Olig2 antibody. From the manufacturer: Species reactivity: mouse, human rat, recognizes Oligodendrocyte lineage transcription factor 2 (Olig2)
(https://www.emdmillipore.com/US/en/product/Anti-Olig2-Antibody-clone-211F1.1,MM_NF-MABN50)

Anti-Flag antibody. From the manufacturer: The ANTI-FLAG M2 mouse, affinity purified monoclonal antibody binds to fusion proteins containing a FLAG peptide sequence.The antibody recognizes the FLAG peptide sequence at the N-terminus, Met-N-terminus,C-terminus, and internal sites of the fusion protein. For highly sensitive and specific detection of FLAG fusion proteins by immunoblotting, (https://www.sigmaaldrich.com/catalog/product/sigma/f1804?lang=en&region=US). (Arne Jahn et. al., 2017, EMBO reports, 18(6), 929-946, 2017-5-14). Also, in our manuscript, Fig. 4h, the anti-flag antibody detected flag-tagged Igfbp2 in all the lanes of cells that were transfected with flag-tagged Igfbp2 plasmids but not the endogenous Igfbp2 in the last lane in the blot.

Anti-Igfbp2 rabbit polyclonal antibody. From the manufacturer's data-sheet as well as the antibody registry: Recommended for mmunocytochemistry; Immunoprecipitation; Western Blot. Recognizes bovine, human, cow, pig, goat, horse, mouse, rat and other primate Igfbp-2 (https://antibodyregistry.org/search.php?q=AB_310049). (Miraki-Mond et al., Clinical Endocrinology. 53: 1-11, 2000).

Anti-Igfbp2 goat polyclonal antibody. From the manufacturer: Detects mouse IGFBP-2 in direct ELISAs and Western blots. In direct ELISAs, approximately 35% cross-reactivity with recombinant human (rh) IGFBP-2 is observed and less than 1% cross-reactivity with rhIGFBP-1, rhIGFBP-3, rhIGFBP-4, rhIGFBP-5, recombinant mouse (rm) IGFBP-3, rmIGFBP-5, and rmIGFBP-6 is observed. (https://www.rndsystems.com/products/mouse-igfbp-2-antibody_af797). (Fletcher et al., 2013, BMC Neurosci, 2013;14(0):158).

Anti-gamma H2A.X (phospho S139) antibody. From the manufacturer: Tested applications: IP, IHC-P, WB, ICC/IF, Dot blot. Species reactivity: reacts with Mouse, Rat, Human. (https://www.abcam.com/gamma-h2ax-phospho-s139-antibody-ep8542y-ab81299.html). (Mangeot et al., 2019, Nat Commun 10:45).

Anti-Gapdh antibody. From the manufacturer: This Antibody was verified by Knockdown to ensure that the antibody binds to the antigen stated. Antibody specificity was demonstrated by siRNA mediated knockdown of target protein. HeLa cells were transfected with GAPDH siRNA and loss of signal was observed in Western Blot using Anti- GAPDH Polyclonal Antibody. (https://www.thermofisher.com/antibody/product/GAPDH-Antibody-Polyclonal/PA1-9046). (Hintze et al., 2020, Frontiers in physiology).

Anti-Rad51 antibody. From the manufacturer: Tested applications: WB, IHC-P, ICC/IF. Reacts with: Mouse, Rat, Human. (https://www.abcam.com/rad51-antibody-bsa-and-azide-free-ab88572.html). (Zhang et al., 2015, Int J Nanomedicine 10:1335-57), (Boysen et al., 2015, Elife 4:N/A).

Animals and other organisms

Policy information about studies involving animals; ARRIVE guidelines recommended for reporting animal research

Laboratory animals	This study used mice either C57BL/6J, Ncf1 KO and Igfbp2 KO mice. Animals of both sex were used in these studies and all animals were at least 8 weeks of age prior to starting the procedures, except for preparation of neonatal NSCs, where we used 0-2 day old mouse pups. Animals were typically group housed and maintained in 12 hour light/dark cycle with set points at 73 degrees Fahrenheit and 50 % humidity.
Wild animals	Study did not involve wild animals
Field-collected samples	Study did not involve field collected samples
Ethics oversight	The University of Iowa IACUC approved all protocols prior to commencing these studies

Note that full information on the approval of the study protocol must also be provided in the manuscript.
