# Supplementary material for: Redox-dependent Igfbp2 signaling controls Brca1 DNA damage response to govern neural stem cell fate
Source: Nat Commun. 2023 Jan 27;14:444. doi: 10.1038/s41467-023-36174-z (PMC9883463; doi:10.1038/s41467-023-36174-z)
Supplement: Supplementary file 7 — Supplementary Software 1 [file 41467_2023_36174_MOESM7_ESM.zip › expected_outputs.zip!/outputs/ST_RNAseq_analysis.pdf]

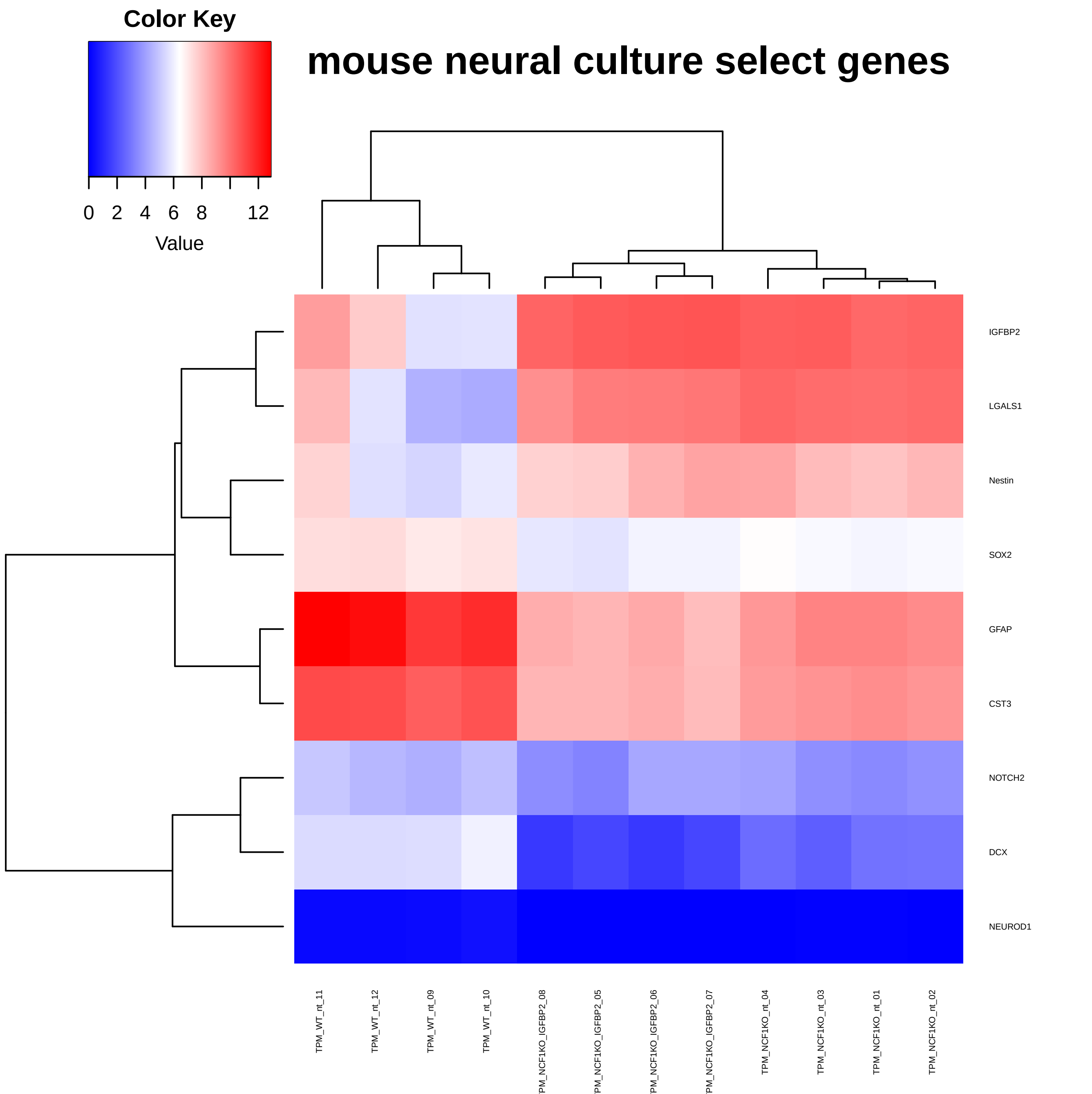

Color Key
0 2 4 6 8 12
Value
mouse neural culture select genes
IGFBP2
LGALS1
Nestin
SOX2
GFAP
CST3
NOTCH2
DCX
NEUROD1
TPM_WT_nt_11
TPM_WT_nt_12
TPM_WT_nt_09
TPM_WT_nt_10
PM_NCF1KO_IGFBP2_08
PM_NCF1KO_IGFBP2_05
PM_NCF1KO_IGFBP2_06
PM_NCF1KO_IGFBP2_07
TPM_NCF1KO_nt_04
TPM_NCF1KO_nt_03
TPM_NCF1KO_nt_01
TPM_NCF1KO_nt_02

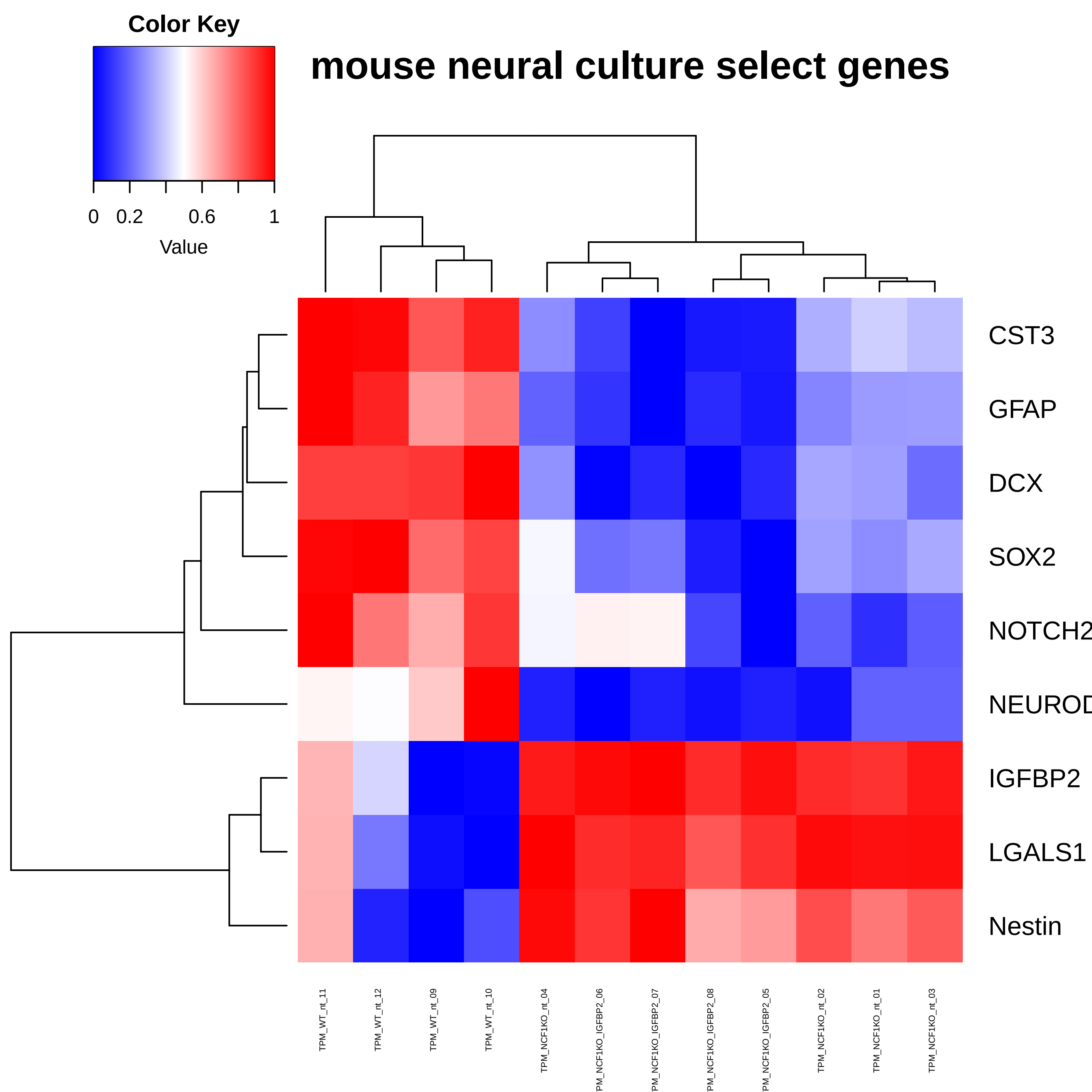

Color Key
0 0.2 0.6 1
Value
mouse neural culture select genes
CST3
GFAP
DCX
SOX2
NOTCH2
NEUROD
IGFBP2
LGALS1
Nestin
TPM_WT_nt_11
TPM_WT_nt_12
TPM_WT_nt_09
TPM_WT_nt_10
TPM_NCF1KO_nt_04
PM_NCF1KO_IGFBP2_06
PM_NCF1KO_IGFBP2_07
PM_NCF1KO_IGFBP2_08
PM_NCF1KO_IGFBP2_05
TPM_NCF1KO_nt_02
TPM_NCF1KO_nt_01
TPM_NCF1KO_nt_03

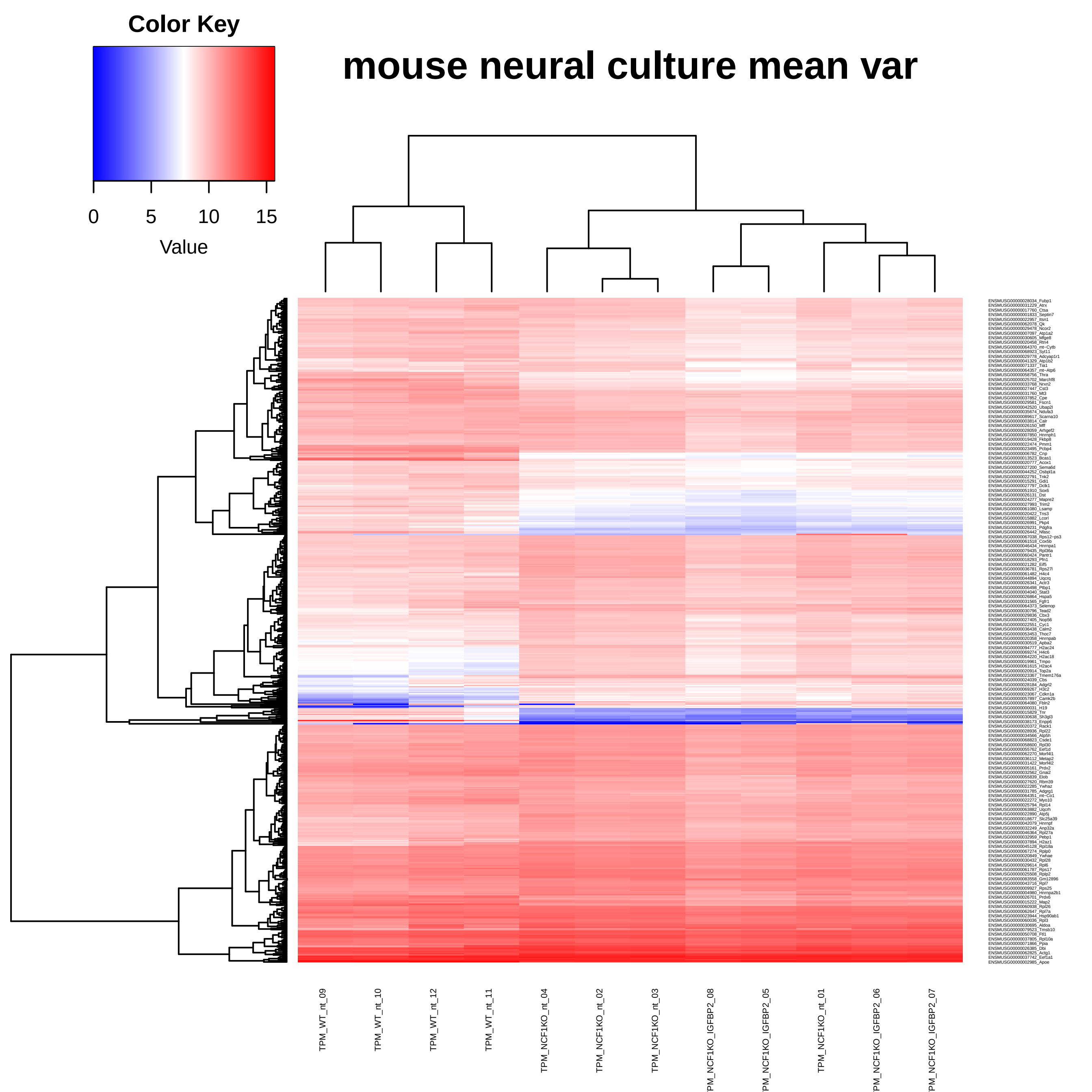
Color Key
0
5
10
15
Value
mouse neural culture mean var
TPM_WT_nt_09
TPM_WT_nt_10
TPM_WT_nt_12
TPM_WT_nt_11
TPM_NCF1KO_nt_04
TPM_NCF1KO_nt_02
TPM_NCF1KO_nt_03
PM_NCF1KO_IGFBP2_08
PM_NCF1KO_IGFBP2_05
TPM_NCF1KO_nt_01
PM_NCF1KO_IGFBP2_06
PM_NCF1KO_IGFBP2_07

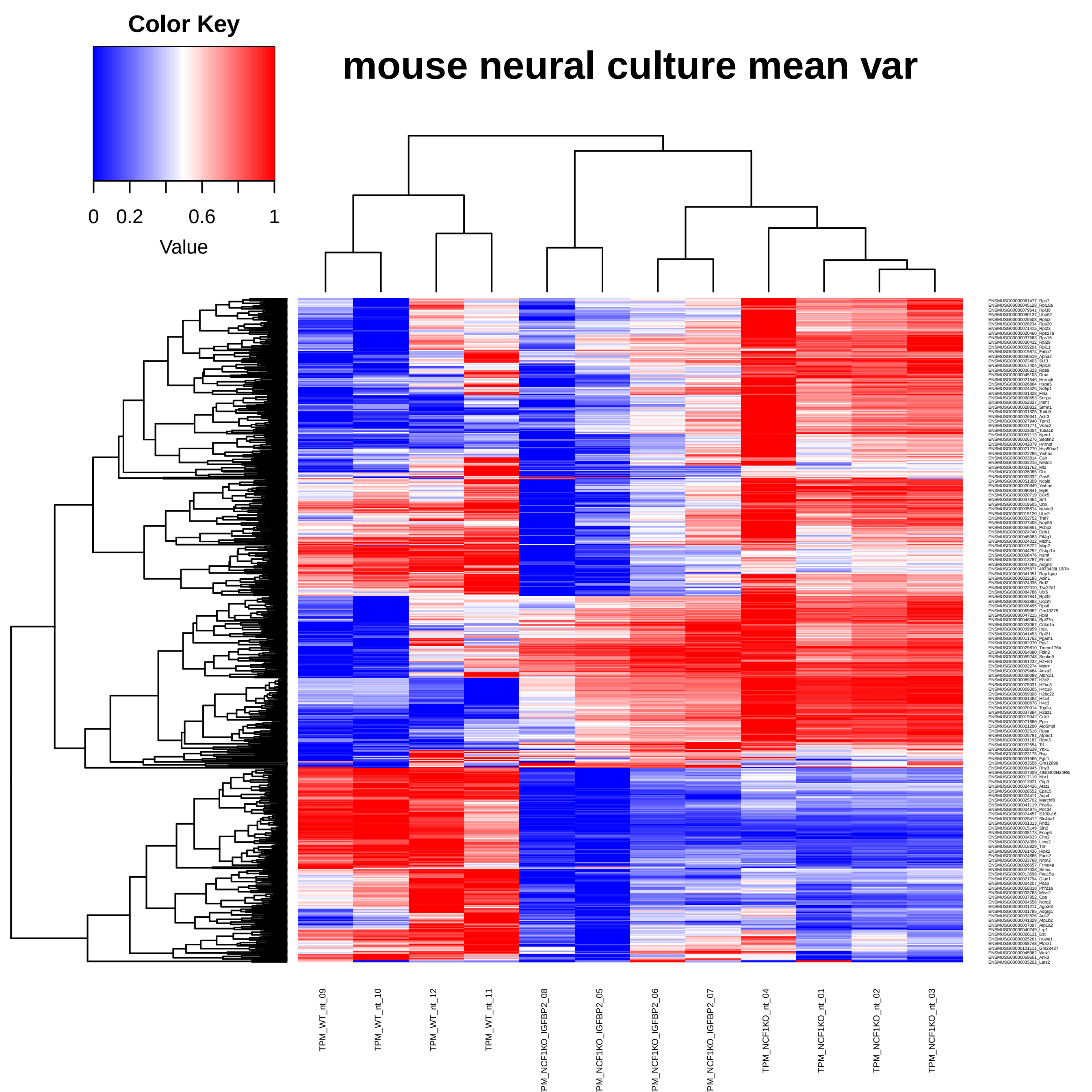
mouse neural culture mean var
Color Key
0
0.2
0.6
1
Value
TPM_WT_nt_09
TPM_WT_nt_10
TPM_WT_nt_12
TPM_WT_nt_11
PM_NCF1KO_IGFBP2_08
PM_NCF1KO_IGFBP2_05
PM_NCF1KO_IGFBP2_06
PM_NCF1KO_IGFBP2_07
TPM_NCF1KO_nt_04
TPM_NCF1KO_nt_01
TPM_NCF1KO_nt_02
TPM_NCF1KO_nt_03

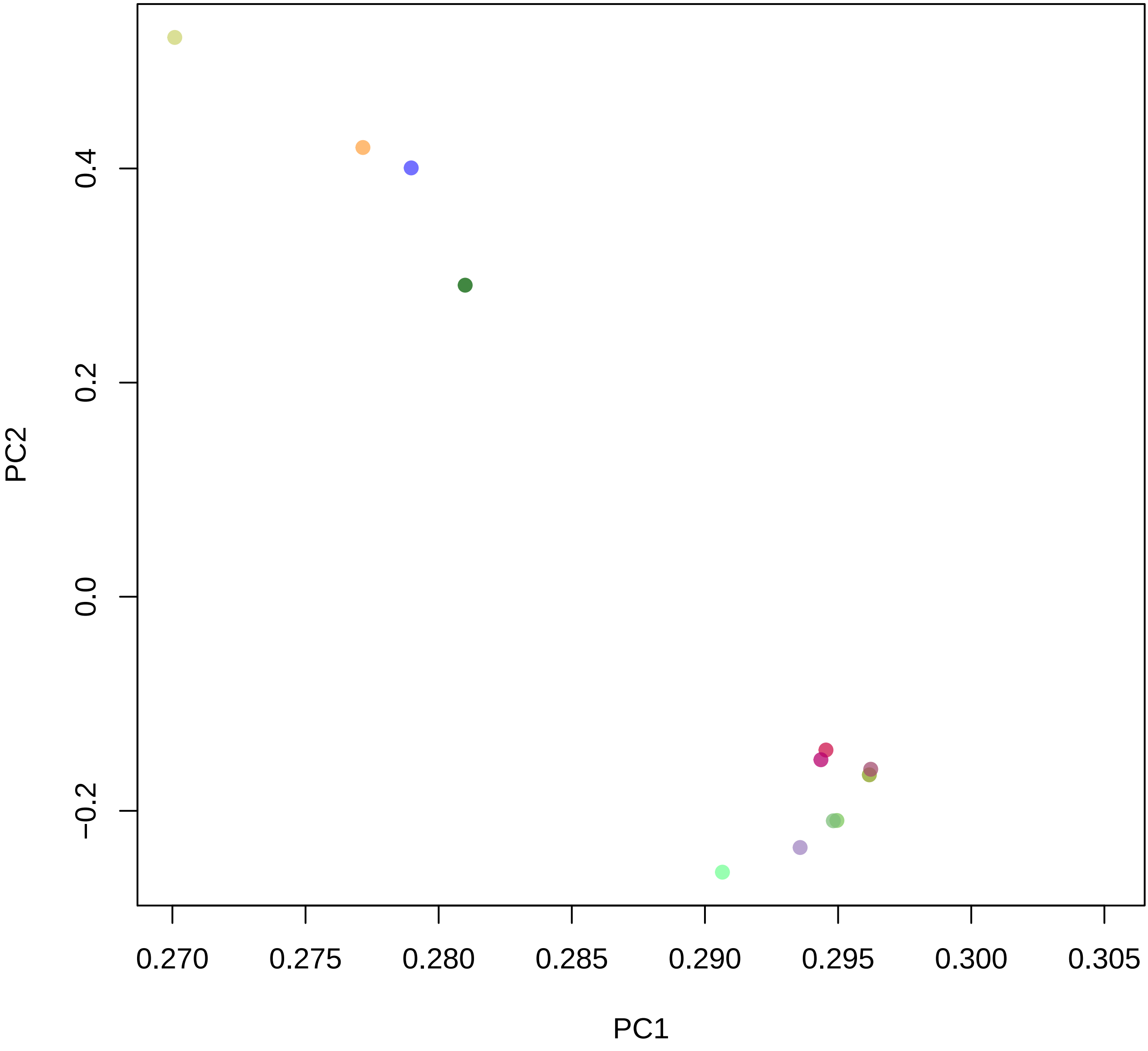
PC2
0.4
0.2
0.0
−0.2
0.270
0.275
0.280
0.285
0.290
0.295
0.300
0.305
PC1

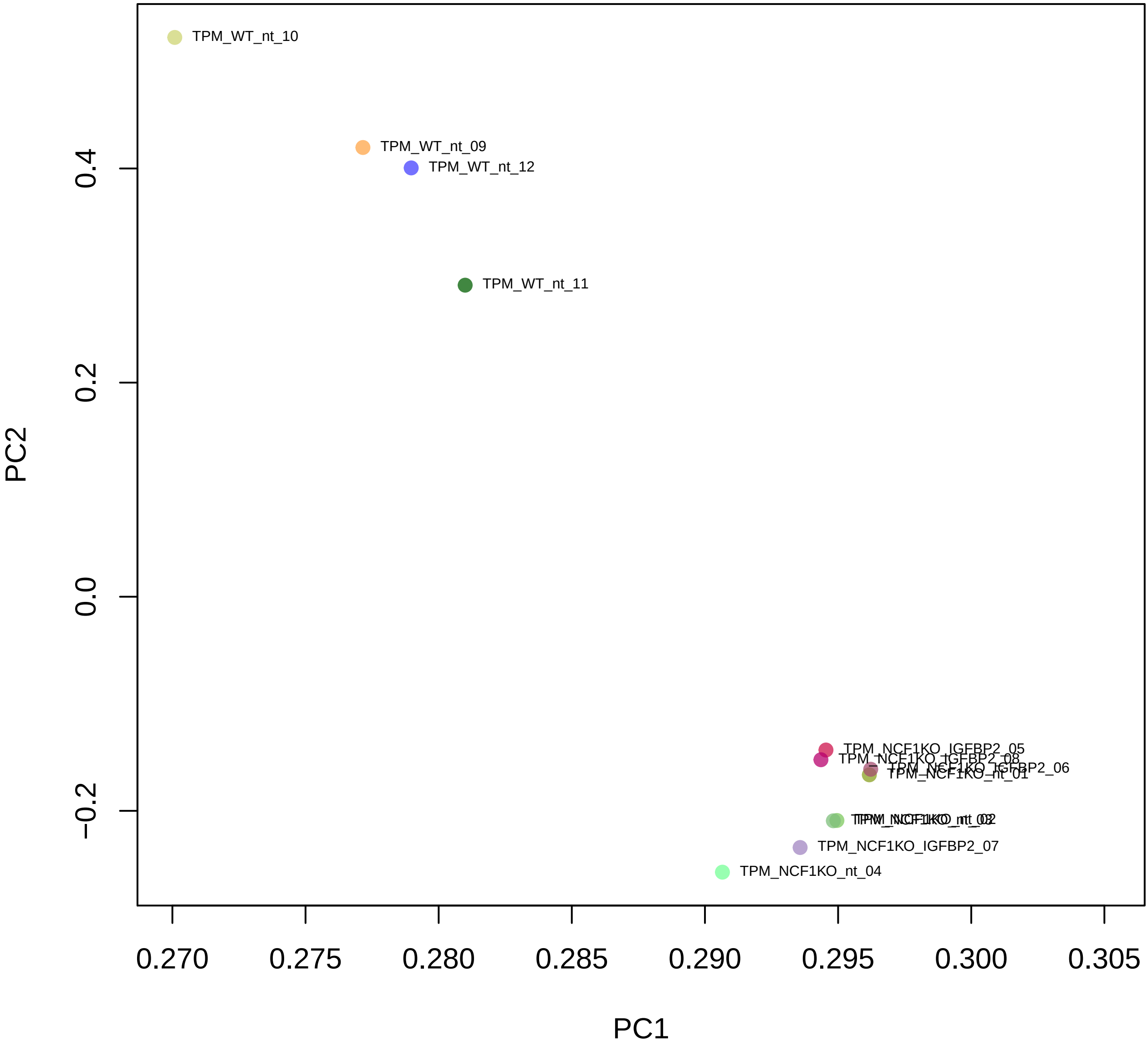

TPM_WT_nt_10
TPM_WT_nt_09
TPM_WT_nt_12
TPM_WT_nt_11
TPM_NCF1KO_IGFBP2_05
TPM_NCF1KO_IGFBP2_08
TPM_NCF1KO_IGFBP2_06
TPM_NCF1KO_nt_01
TPM_NCF1KO_IGFBP2_07
TPM_NCF1KO_nt_04
0.4
0.2
0.0
−0.2
PC2
0.270
0.275
0.280
0.285
0.290
0.295
0.300
0.305
PC1

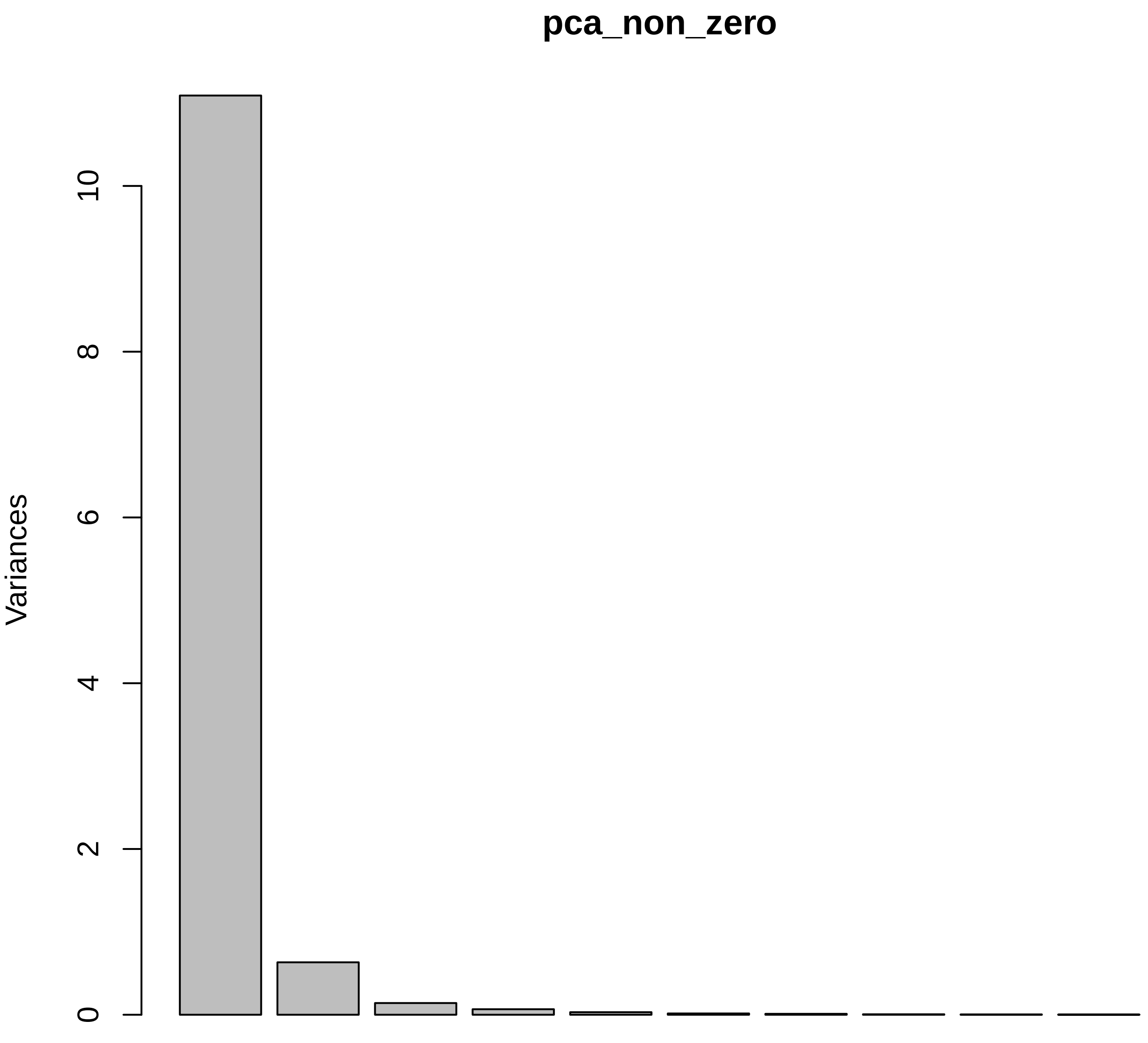
pca_non_zero
Variances
0
2
4
6
8
10

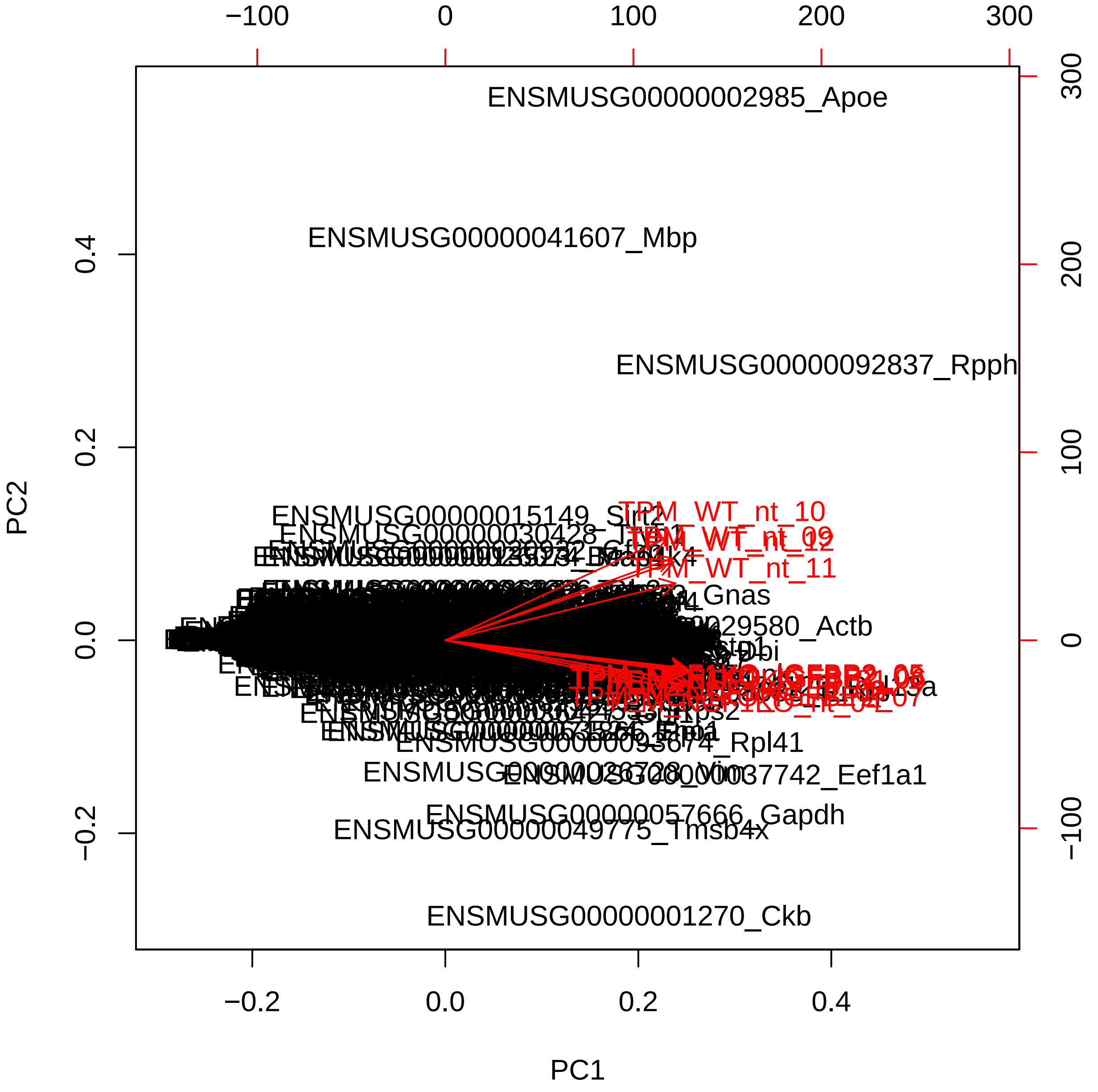

−100
0
100
200
300
ENSMUSG00000002985_Apoe
ENSMUSG00000041607_Mbp
ENSMUSG00000092837_Rpph
ENSMUSG00000015149_Sirt2
TPM_WT_nt_10
TPM_WT_nt_11
ENSMUSG00000093674_Rpl41
ENSMUSG00000037742_Eef1a1
ENSMUSG00000057666_Gapdh
ENSMUSG00000049775_Tmsb4x
ENSMUSG00000001270_Ckb
0.4
0.2
0.0
−0.2
PC2
−0.2
0.0
0.2
0.4
PC1

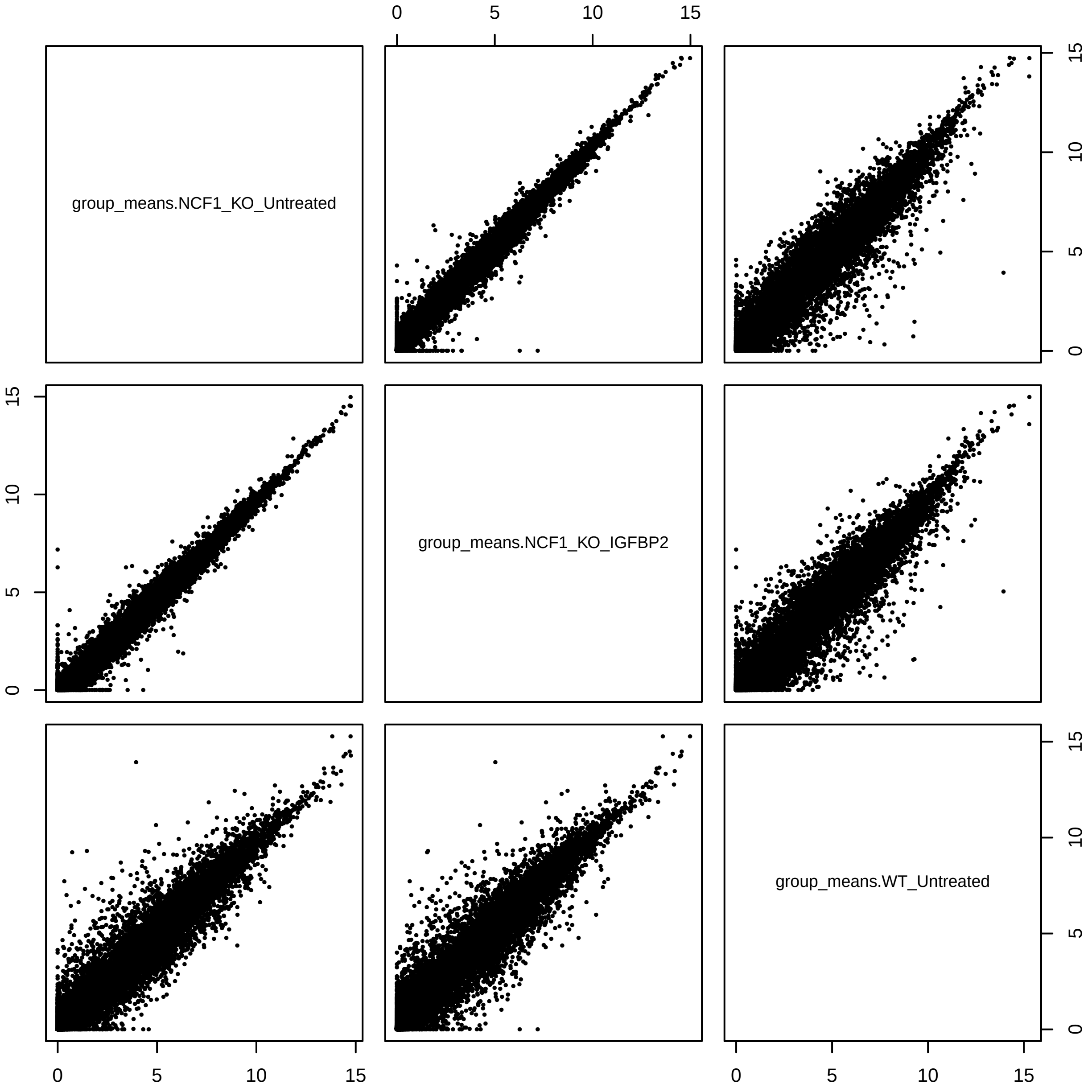
group_means.NCF1_KO_Untreated
group_means.NCF1_KO_IGFBP2
group_means.WT_Untreated
0
5
10
15

Histogram of na.omit(final_summary_stats_df[, "kw"])

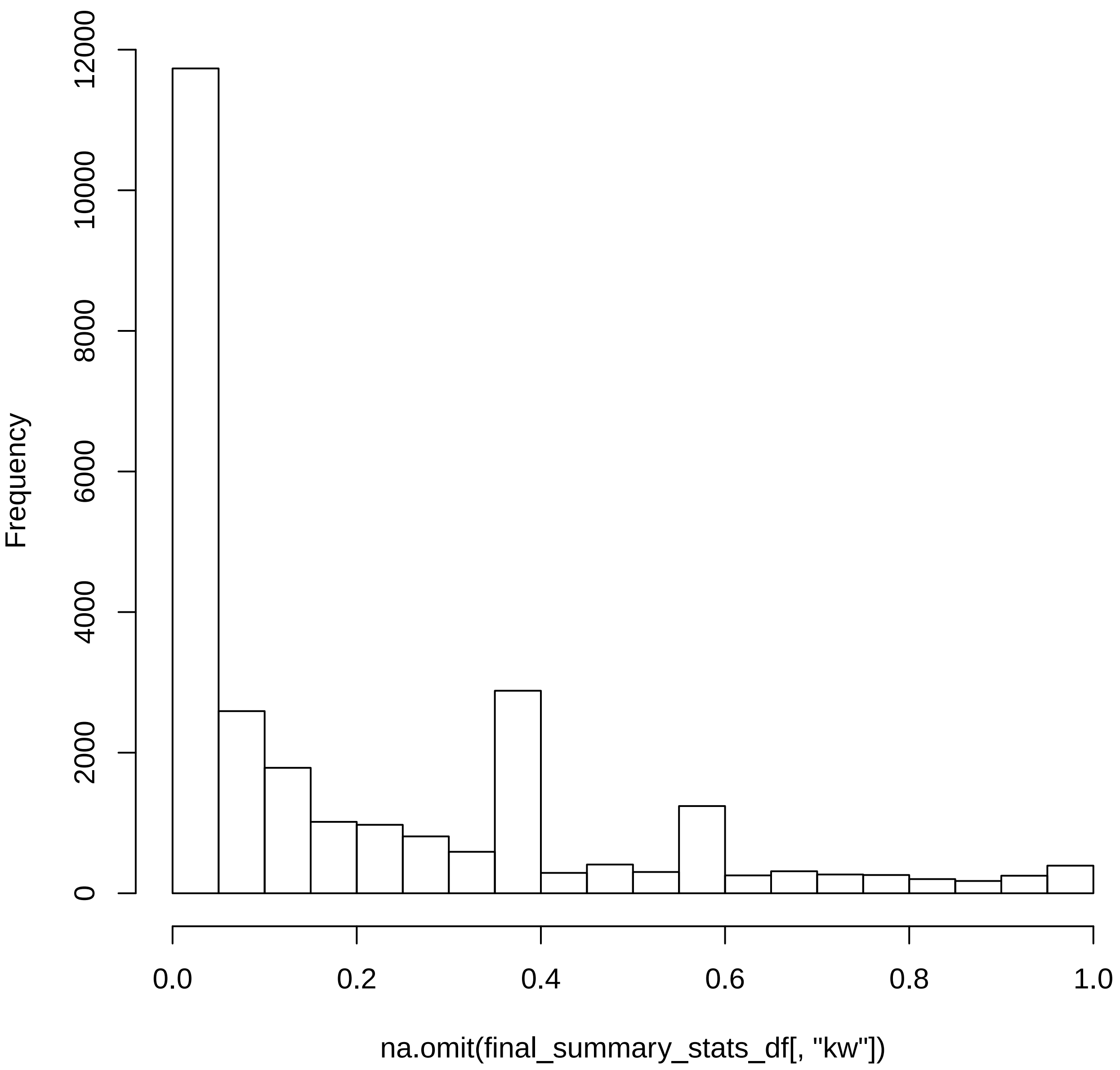

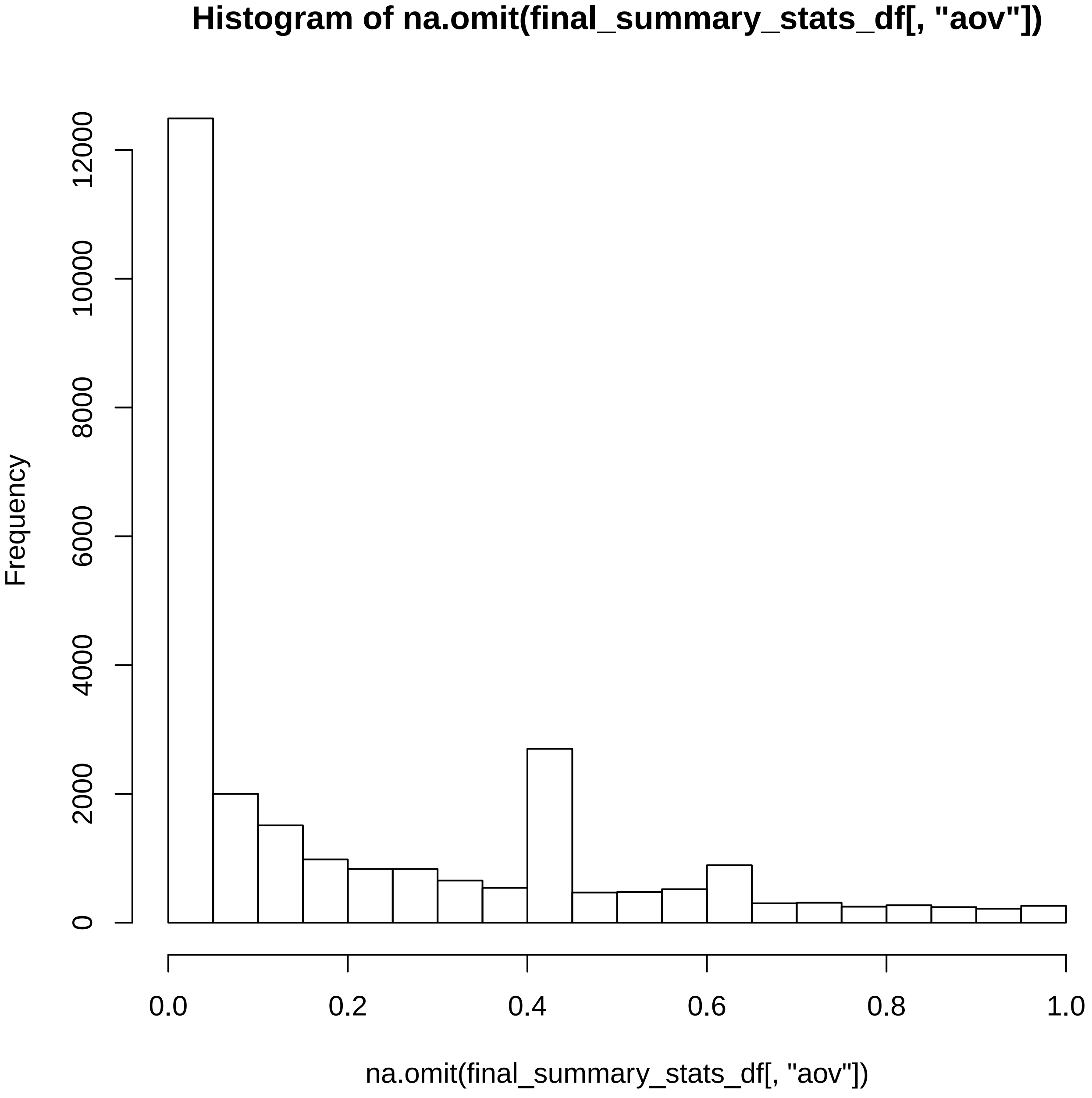
Histogram of na.omit(final_summary_stats_df[, "aov"])
Frequency
0
2000
4000
6000
8000
10000
12000
0.0
0.2
0.4
0.6
0.8
1.0
na.omit(final_summary_stats_df[, "aov"])

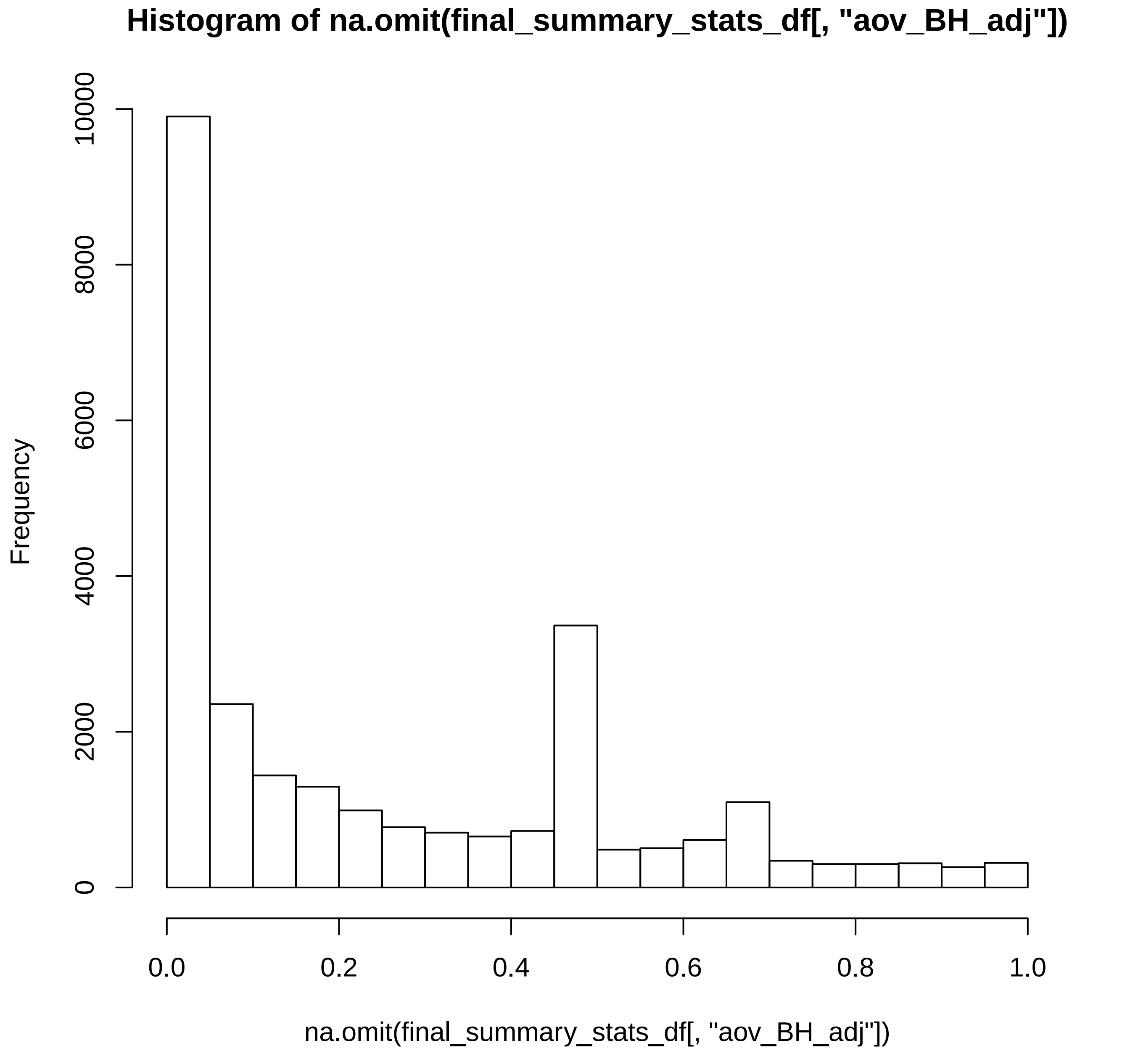

Histogram of na.omit(final_summary_stats_df[, "aov_BH_adj"])
Frequency
0
2000
4000
6000
8000
10000
0.0
0.2
0.4
0.6
0.8
1.0
na.omit(final_summary_stats_df[, "aov_BH_adj"])

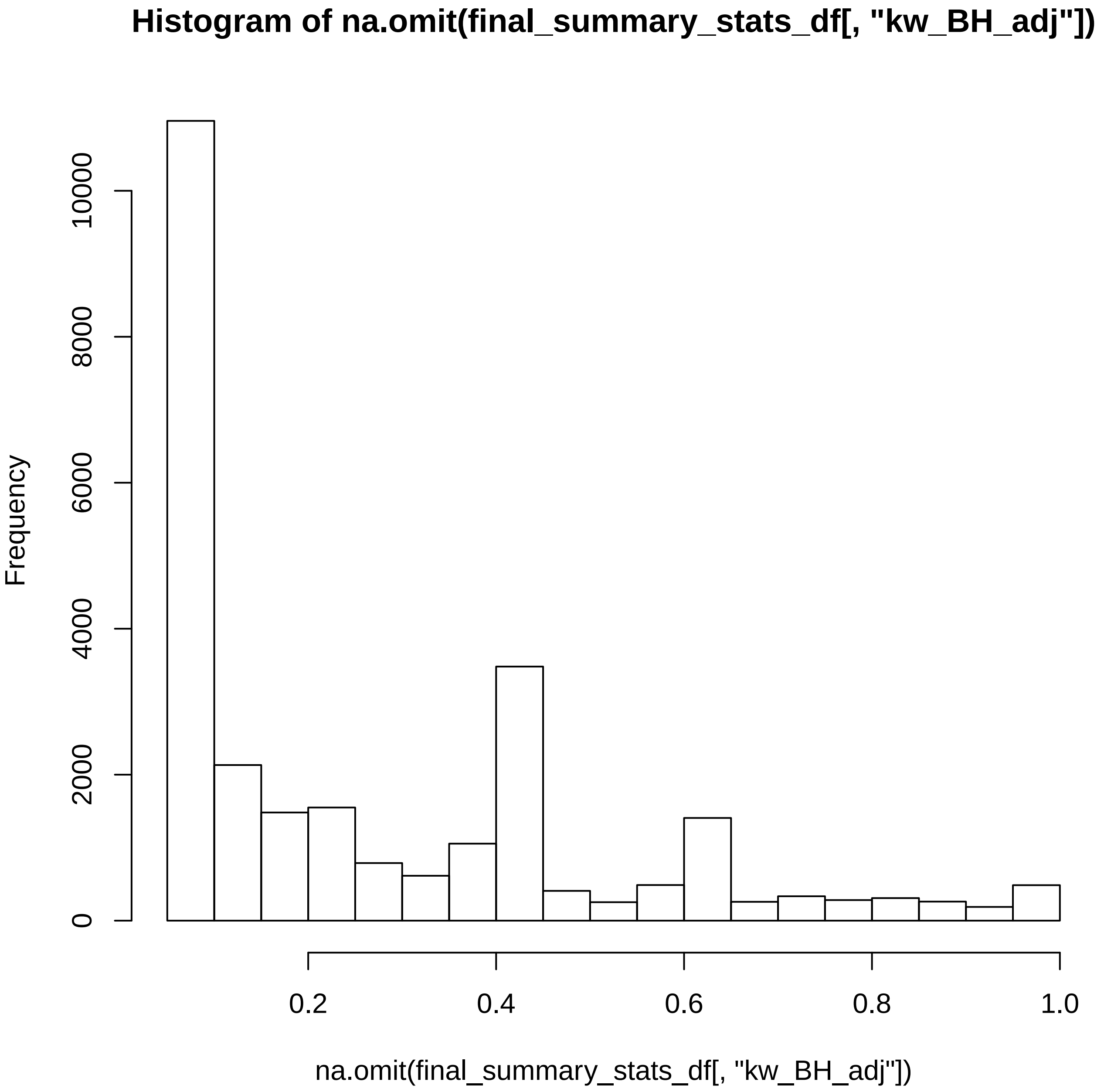

Histogram of na.omit(final_summary_stats_df[, "kw_BH_adj"])
Frequency
0
2000
4000
6000
8000
10000
0.2
0.4
0.6
0.8
1.0
na.omit(final_summary_stats_df[, "kw_BH_adj"])

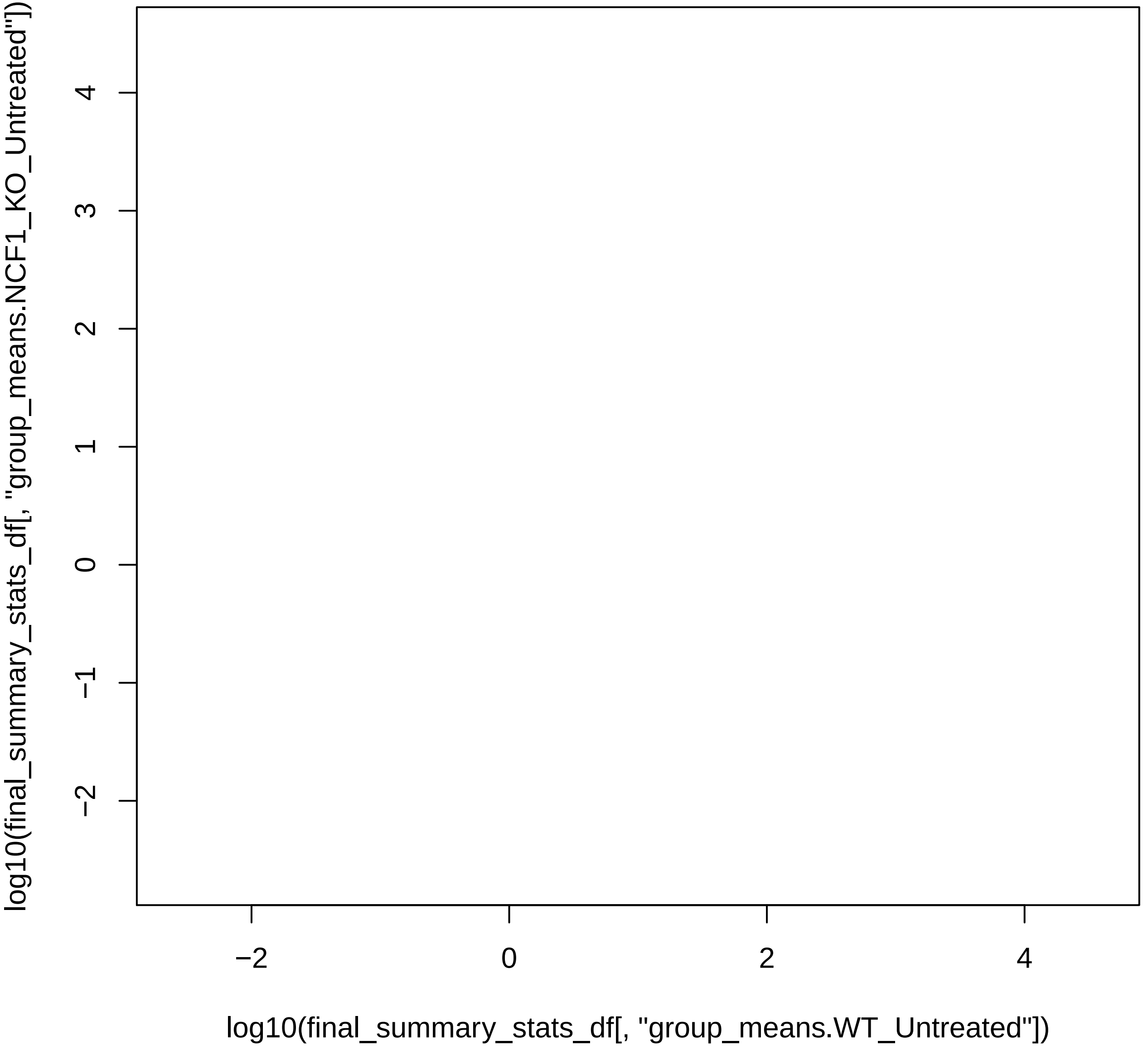
log10(final_summary_stats_df[, "group_means.NCF1_KO_Untreated"])
−2
−1
0
1
2
3
4
−2
0
2
4
log10(final_summary_stats_df[, "group_means.WT_Untreated"])

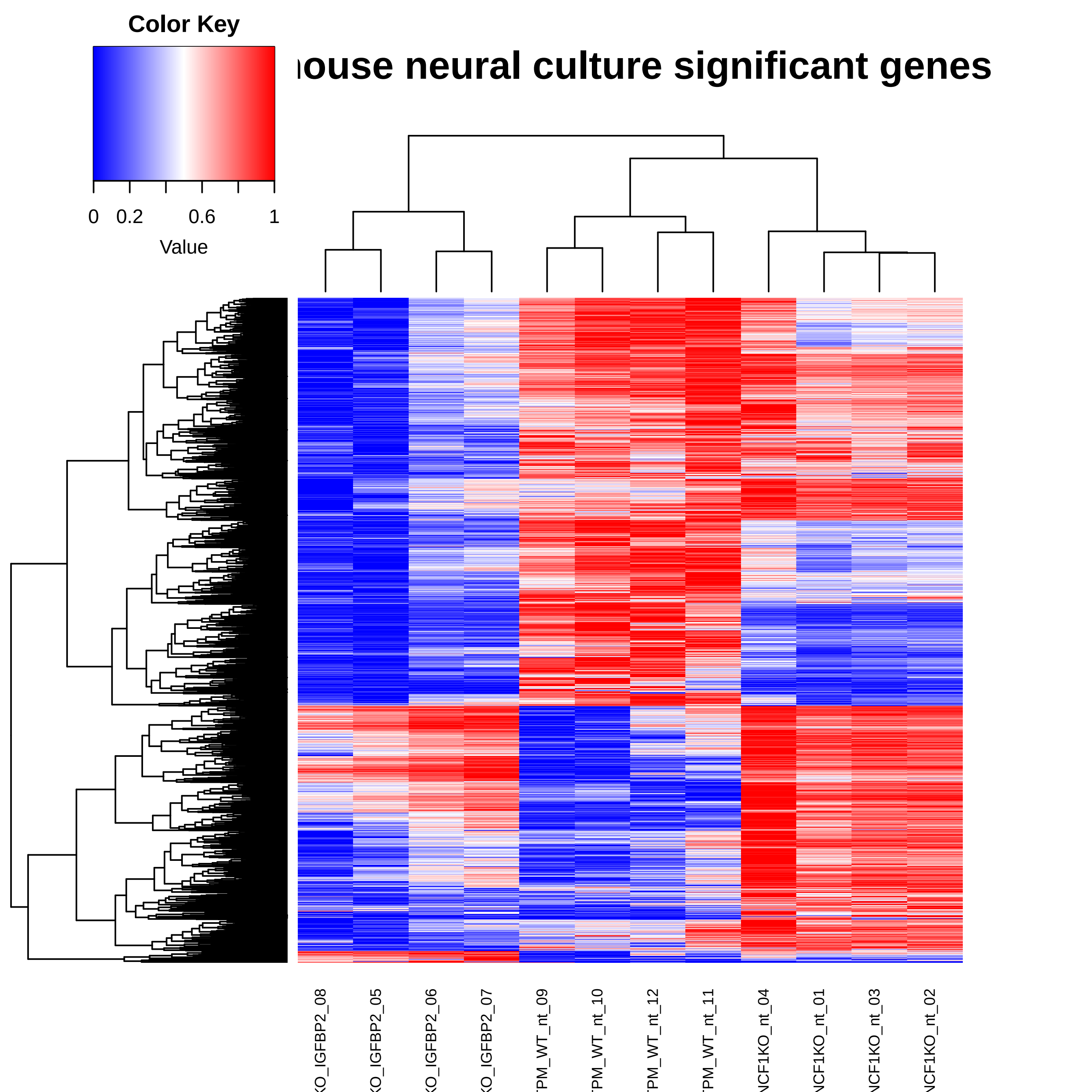
Color Key
0
0.2
0.6
1
Value
ouse neural culture significant genes
KO_IGFBP2_08
KO_IGFBP2_05
KO_IGFBP2_06
KO_IGFBP2_07
TPM_WT_nt_09
TPM_WT_nt_10
TPM_WT_nt_12
TPM_WT_nt_11
NCF1KO_nt_04
NCF1KO_nt_01
NCF1KO_nt_03
NCF1KO_nt_02

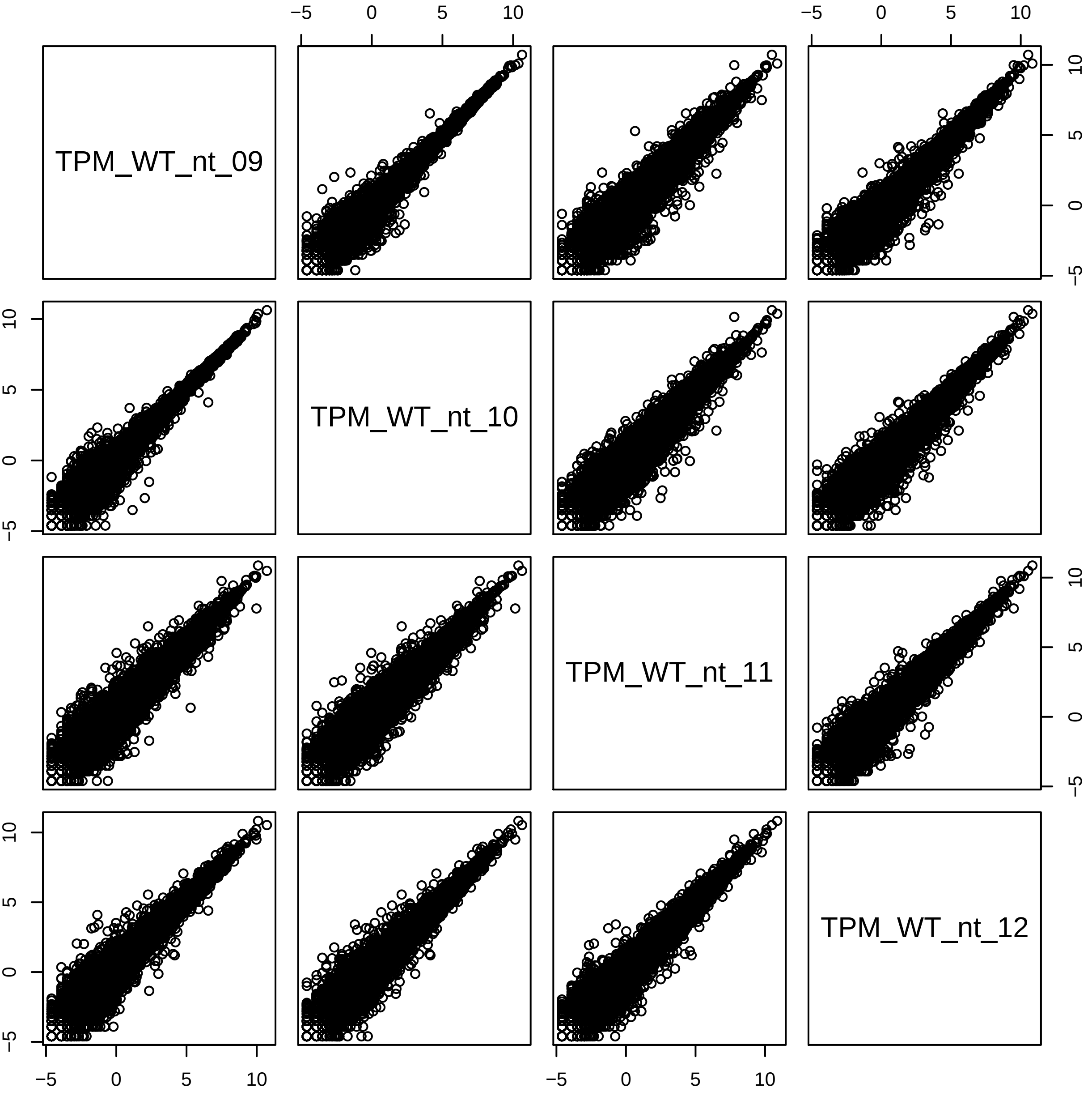
TPM_WT_nt_09
TPM_WT_nt_10
TPM_WT_nt_11
TPM_WT_nt_12
−5
0
5
10

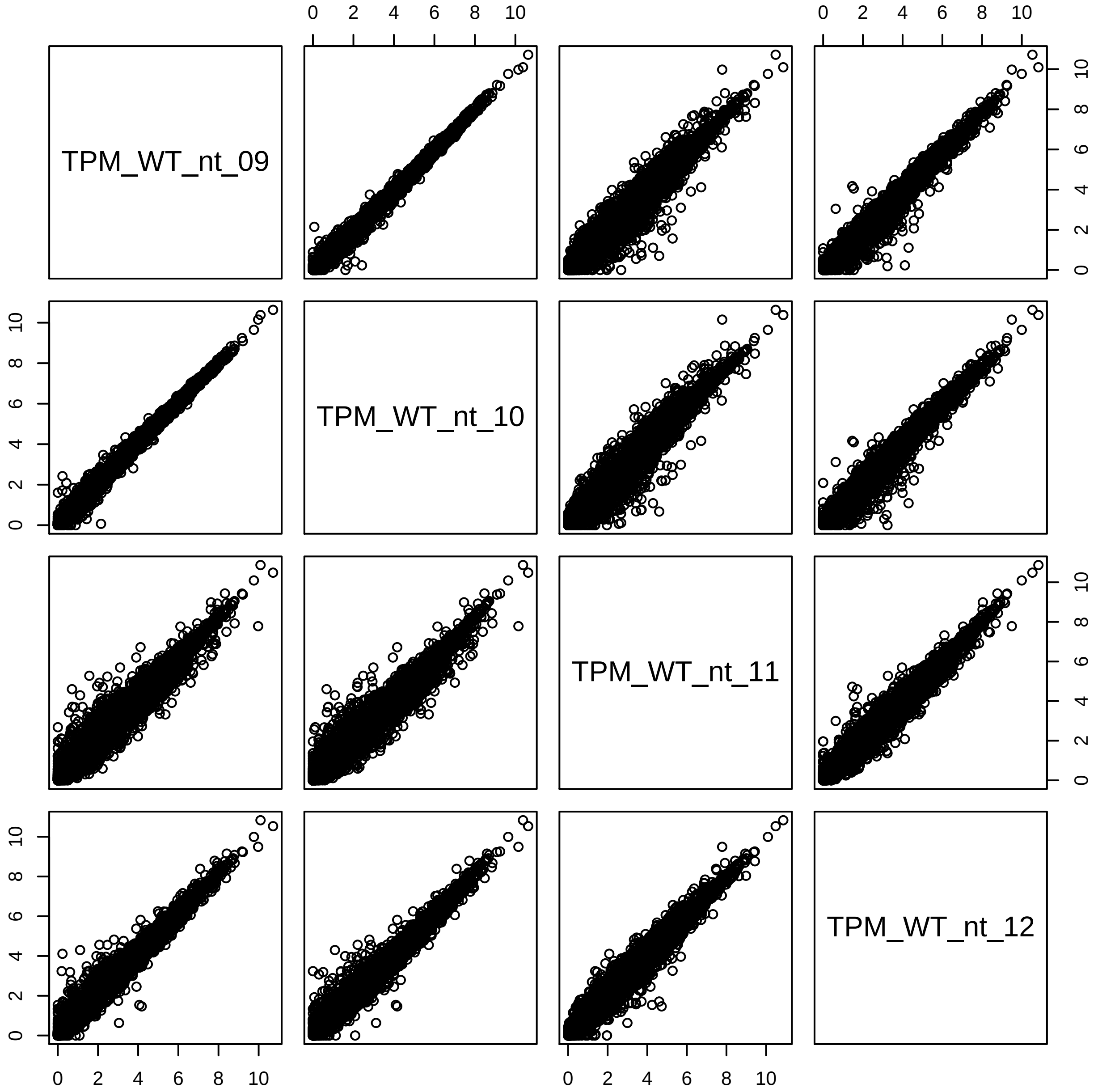

TPM_WT_nt_09
TPM_WT_nt_10
TPM_WT_nt_11
TPM_WT_nt_12

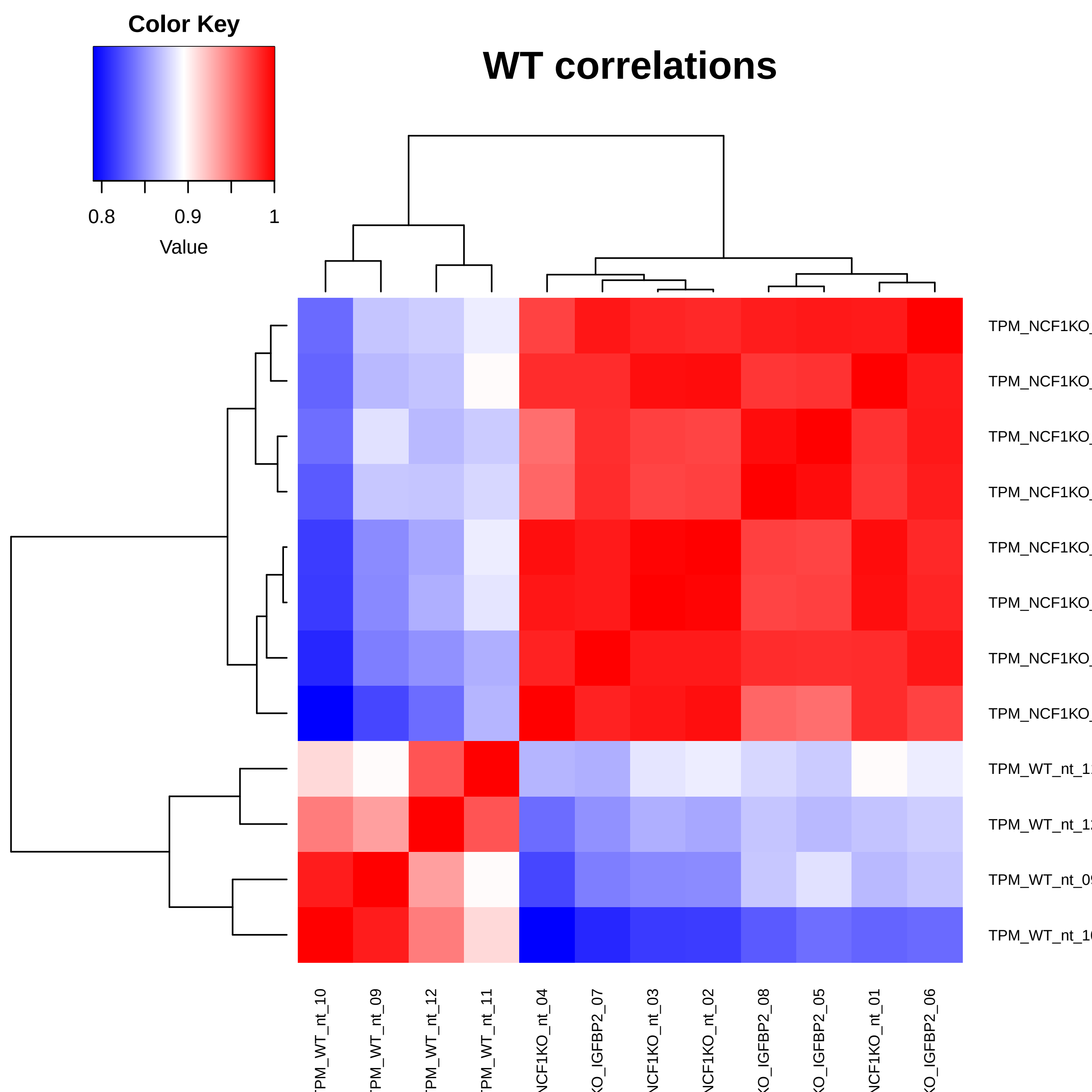
Color Key
0.8
0.9
1
Value
WT correlations
TPM_NCF1KO_
TPM_NCF1KO_
TPM_NCF1KO_
TPM_NCF1KO_
TPM_NCF1KO_
TPM_NCF1KO_
TPM_NCF1KO_
TPM_NCF1KO_
TPM_WT_nt_1
TPM_WT_nt_1
TPM_WT_nt_0
TPM_WT_nt_1
PM_WT_nt_10
PM_WT_nt_09
PM_WT_nt_12
PM_WT_nt_11
NCF1KO_nt_04
KO_IGFBP2_07
NCF1KO_nt_03
NCF1KO_nt_02
KO_IGFBP2_08
KO_IGFBP2_05
NCF1KO_nt_01
KO_IGFBP2_06

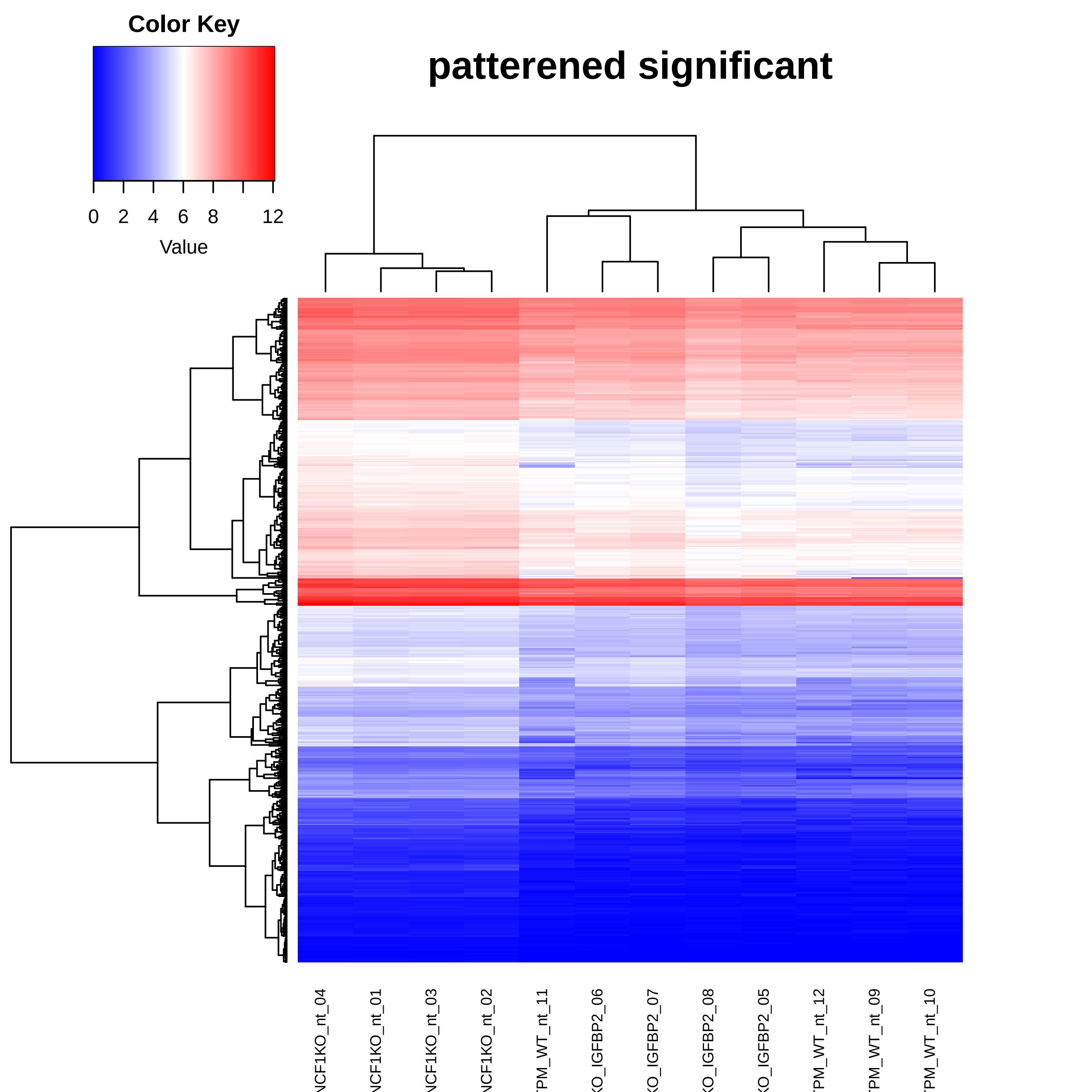

Color Key
0 2 4 6 8 12
Value
patterened significant
NCF1KO_nt_04
NCF1KO_nt_01
NCF1KO_nt_03
NCF1KO_nt_02
TPM_WT_nt_11
KO_IGFBP2_06
KO_IGFBP2_07
KO_IGFBP2_08
KO_IGFBP2_05
TPM_WT_nt_12
TPM_WT_nt_09
TPM_WT_nt_10

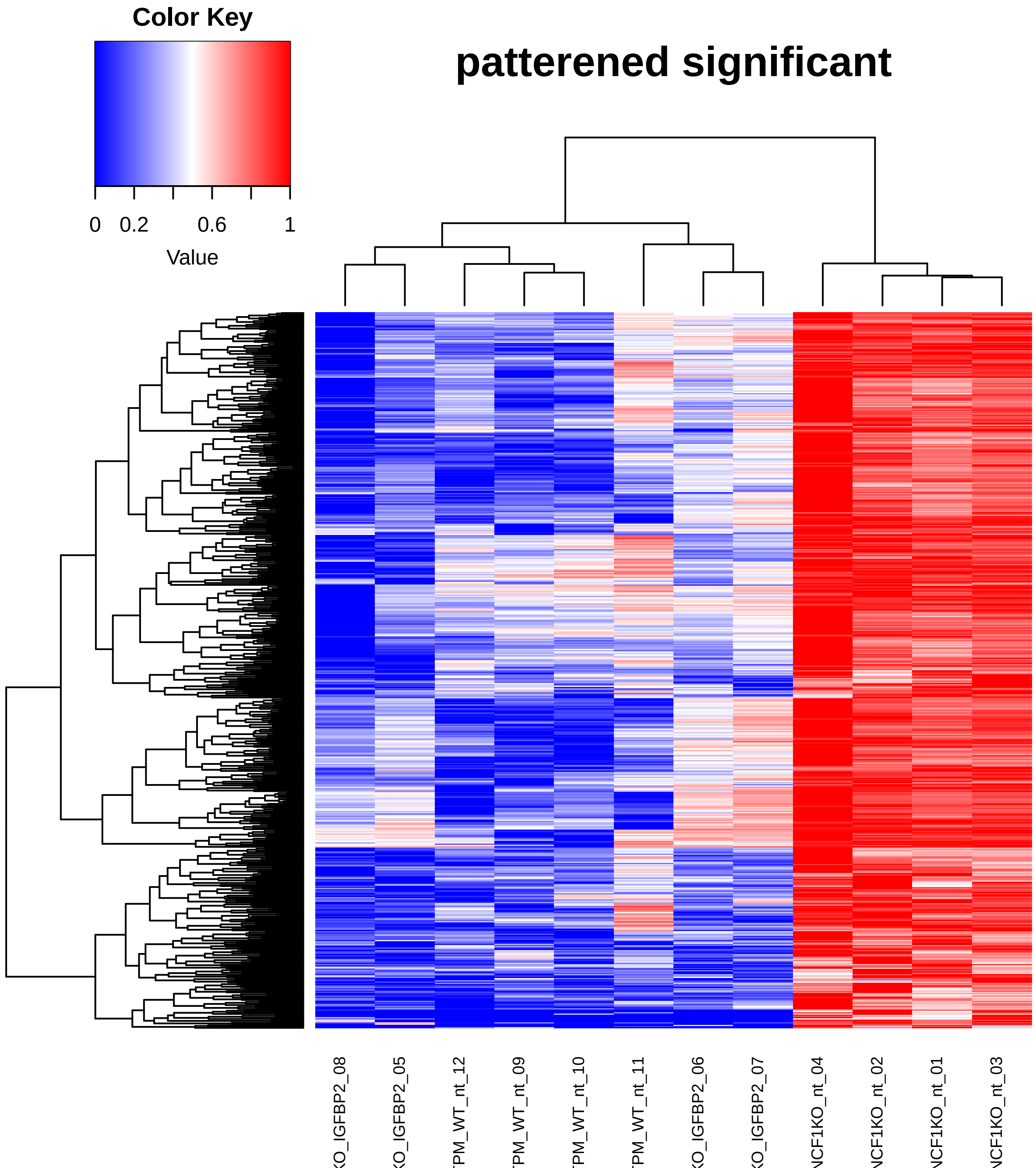
Color Key
0
0.2
0.6
1
Value
patterened significant
KO_IGFBP2_08
KO_IGFBP2_05
TPM_WT_nt_12
TPM_WT_nt_09
TPM_WT_nt_10
TPM_WT_nt_11
KO_IGFBP2_06
KO_IGFBP2_07
NCF1KO_nt_04
NCF1KO_nt_02
NCF1KO_nt_01
NCF1KO_nt_03

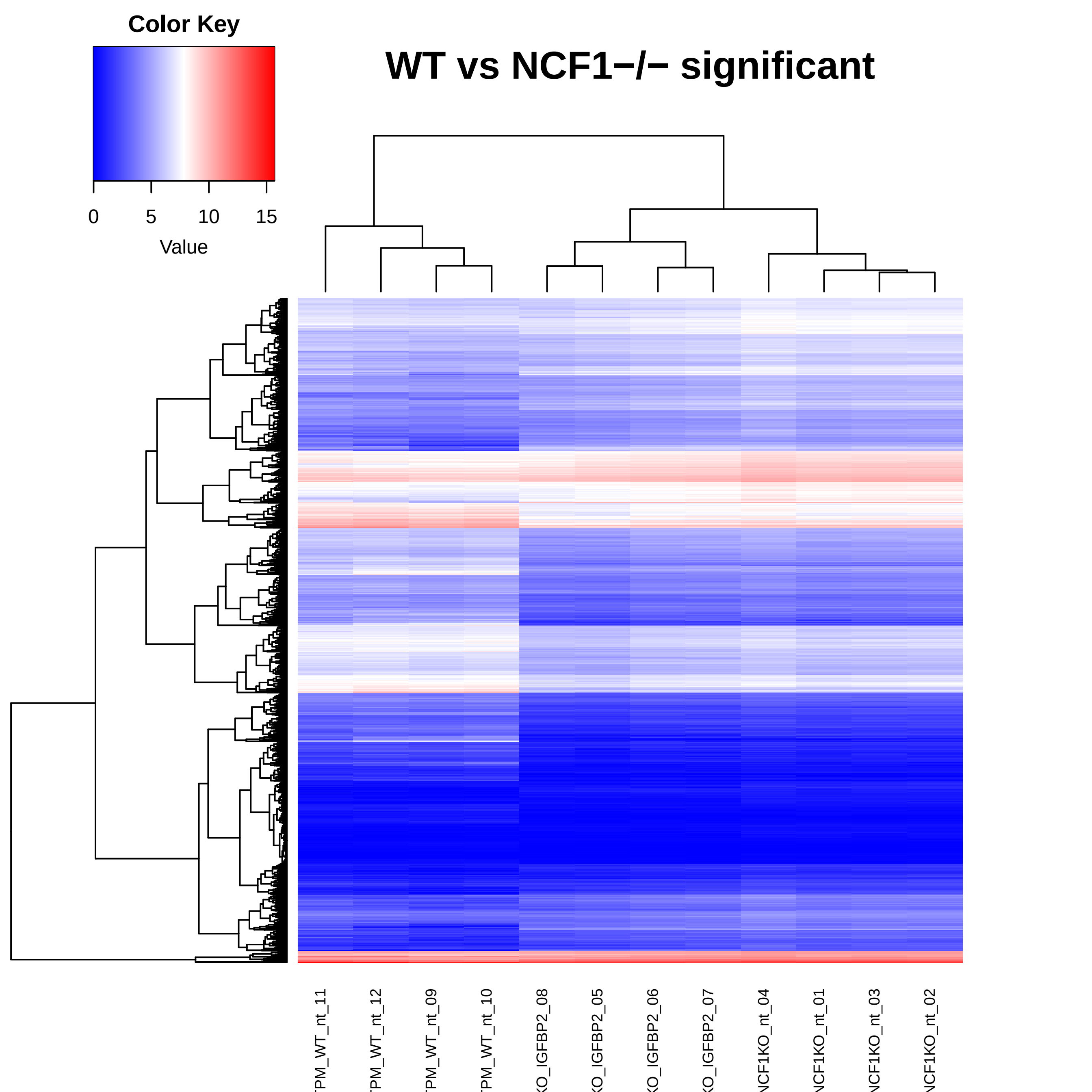

Color Key
0
5
10
15
Value
WT vs NCF1−/− significant
TPM_WT_nt_11
TPM_WT_nt_12
TPM_WT_nt_09
TPM_WT_nt_10
KO_IGFBP2_08
KO_IGFBP2_05
KO_IGFBP2_06
KO_IGFBP2_07
NCF1KO_nt_04
NCF1KO_nt_01
NCF1KO_nt_03
NCF1KO_nt_02

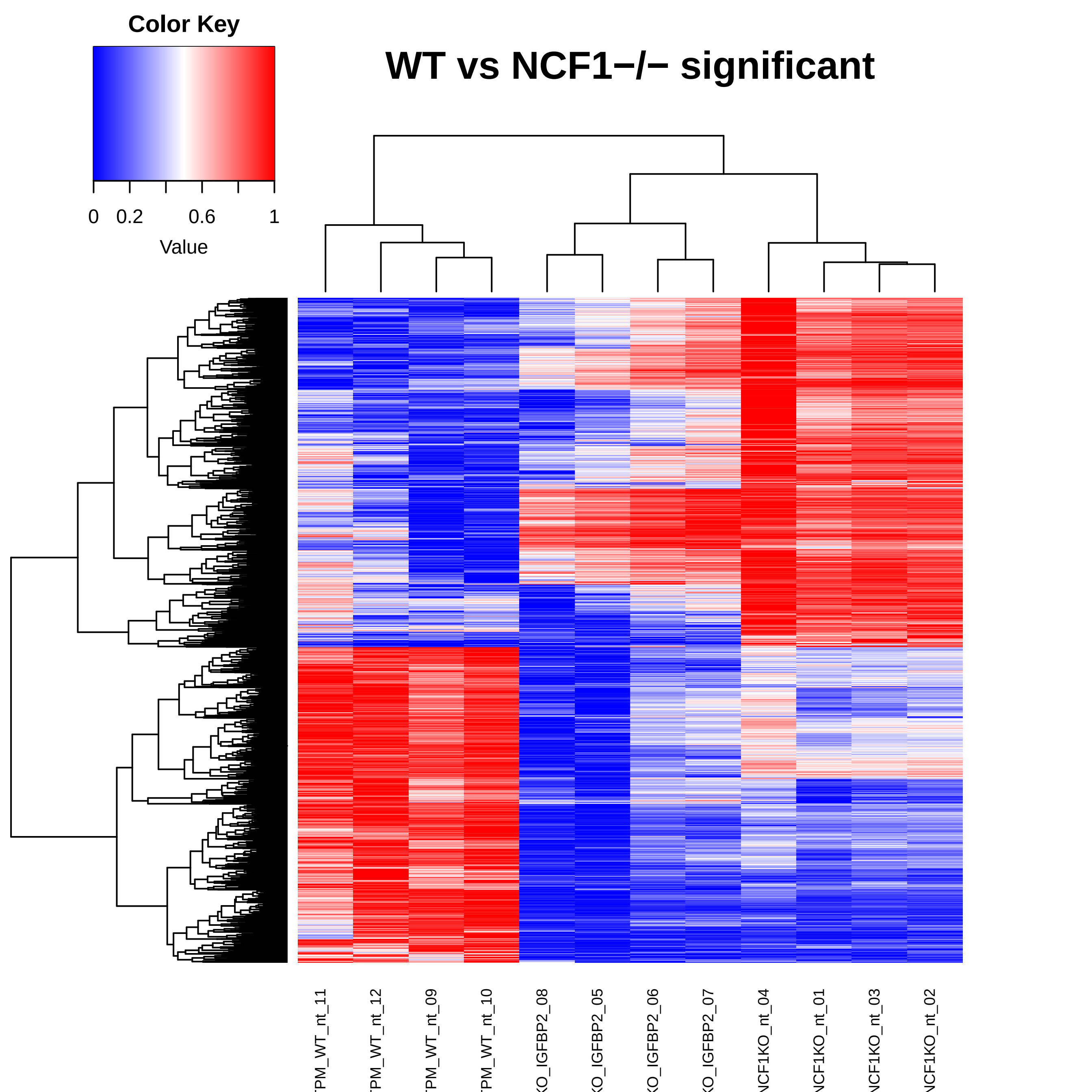
Color Key
0
0.2
0.6
1
Value
WT vs NCF1−/− significant
WT_nt_11
WT_nt_12
WT_nt_09
WT_nt_10
IGFBP2_08
IGFBP2_05
IGFBP2_06
IGFBP2_07
CF1KO_nt_04
CF1KO_nt_01
CF1KO_nt_03
CF1KO_nt_02
